# Genotype by environment interactions and phenotypic traits stability of the EUCLEG faba bean collection

**DOI:** 10.3389/fpls.2024.1480110

**Published:** 2025-01-29

**Authors:** Dejan Sokolović, Snežana Babić, Mirjana Petrović, Ignacio Solís, Mathias Cougnon, Natalia Gutierrez, Pertti Pärssinen, Dirk Reheul, Jasmina Radović, Ana M. Torres

**Affiliations:** ^1^ Institute for Forage Crops Kruševac, Kruševac, Serbia; ^2^ Agrovegetal S.A., Sevilla, Spain; ^3^ Plant Department, ILVO (Flanders Research Institute for Agriculture, Fisheries and Food), Melle, Belgium; ^4^ Área de Mejora Vegetal y Biotecnología, The Andalusian Institute of Agricultural and Fisheries Research and Training (IFAPA) Centro “Alameda del Obispo”, Córdoba, Spain; ^5^ Boreal Plant Breeding Ltd., Jokioinen, Finland; ^6^ Department Plants and Crops, Faculty Bioscience Engineering, Ghent University, Ghent, Belgium

**Keywords:** faba bean, genotype x environment interaction, mega-environments, botanical types, multi-trait stability index, grain legume

## Abstract

Faba bean (*Vicia faba* L.) is an important pulse crop traditionally used for human nutrition and animal feeding. With a high protein content ranging from 24% to 35% of seed dry matter, considerable amounts of globulins, essential amino acids and minerals, faba bean is today an important source meeting the growing global demand for nutritious food. The objective of study was to investigate the variability of nine phenological, phenotypical and yield related traits in 220 faba bean accessions in multi-location trials across four representative European regions. Nine field trials were carried out from 2018 till 2020 in four representative European locations (Spain, Finland, Belgium and Serbia) using an augmented p-rep design containing 20 replicated checks. Significant differences among genotypes and environments were detected, being the genotype x environment interaction (GEI) the major source of variation in five of the nine evaluated traits. The “which-won-where” analyses identified two mega-environment namely South European mega environment (SE-ME) and North European mega environment (NE-ME), while the best performing and most stable genotypes according to the nine traits were identified using “means vs stability” analyses. According to the highest trait value in each mega environment several winning genotypes were identified showing better performances than some commercial varieties (controls) or checks. Our results suggest that the geographical locations falling into each mega-environment can be used as faba bean test locations. The genotype ranking for the multi-trait stability index (MTSI) revealed that the most stable and best ranking genotypes in SE-ME are G018, G086, G081, G170 and G015 while in the north mega-environment are G091, G171, G177 (Merkur), G029 and G027. Hierarchical cluster analysis and principal component analyses showed a clear correlation between the traits analysed and the botanical type. These findings indicate that botanical type is one of the most significant factors affecting development in any environment, and it must be taken into account in faba bean breeding activities. The information derived from this study provides a chance for breeding new resilient faba bean cultivars adapted to different agroecological European regions, a critical point for addressing Europe’s reliance on protein imports and enhancing sustainable agriculture practices.

## 1 Introduction

Legumes are crucial for sustainable agriculture and provide essential plant-based proteins for both human and animal diets. There is an increasing protein demand worldwide higher than 200 million tonnes/year (Henchion et al., 2017) especially in Europe and Asia which are dependent on protein imports. During the last few decades, Europe has imported about 65% of its protein (De Visser et al., 2014) and is now exploring possibilities to further develop protein production in an economically and environmentally sound way (https://eur-lex.europa.eu)^1^.

Faba bean (*Vicia faba* L.) is a major food and feed legume with high protein content (24–30%) (Crépon et al., 2010) well adapted to most climatic areas. With a global harvested area of 2.68 million hectares and a production over 6.14 million metric tons per year (FAOSTAT, 2022) faba bean is today the fourth grain legume in the world just behind pea, chickpea and lentil. Despite the severe decline in the faba bean cultivation witnessed in Europe throughout the 20^th^ century, statistical data shows that figures have started to recover and nowadays represents almost 30% of world faba bean production. Similarly, in China, the largest faba bean worldwide producer (more than 32%), after constant and long-lasting reduction of areas and production, figures started to grow in recent years (FAOSTAT, 2021).

Faba bean is a diploid species (2n=2x=12 chromosomes) with one of the largest genomes among legumes of approximately 13,000 Mb (Johnson et al., 1999; Jayakodi et al., 2023) and a partially allogamous mating system. The percentage of outcrossing varies from about few percent to almost total allogamy (85%) depending on the genotype, the environmental conditions, the pollinator species and their activity at the flowering time (Suso et al., 1999; Maalouf et al., 2002). The evolution of the domesticated faba bean ran along with the proliferation of different seed sizes and shapes, various levels of allogamy and differential winter tolerance. According to the seed size there are four main faba bean botanical types: major (> 800 mg), equina (500–800 mg), minor (< 500 mg) and paucijuga (< 250 mg) (Pietrzak et al., 2016; Stoddard, 2017).The crop is regularly sown in spring, in northern latitudes and in winter in warmer climates, covering a latitudinal range from about 50°N to 40°S and altitudes from the sea level up to 3000 m (Gnanasambandam et al., 2012).

Thanks to genetic improvement strategies, especially in disease and parasites tolerance, the world average faba bean yield per ha has largely improved today up to 2.1 tha-1 in comparison with the 0.9 tha-1 in the past five decades (FAOSTAT, 2021). Moreover, the use of faba bean in cropping systems reduces weeds and enhances different soil organic and physical properties (Salahin et al., 2013; Adekiya et al., 2017). By symbiosis with *Rhizobium*, faba bean may fix up to 200 kg N ha-1 (Neugschwandtner et al., 2015) thus, improving yield performance of the subsequent crops (Duc et al., 2015). Moreover, intercropping faba bean and cereals has been shown to reduce the incidence of different diseases and pests in the legume crop (Hauggaard-Nielsen et al., 2008; Fernandez-Aparicio et al., 2007).

Despite these well recognized benefits, one of the major faba bean challenges, common with many other legumes, is yield stability whose variation is attributed to genotype by environment interactions (GEI). GEI is an important bottleneck in crop breeding programs as it decreases the heritability and the correlation between phenotypic and genotypic values, slowing down selection progress (Alghamdi et al., 2012; Flores et al., 2013; Abebe et al., 2015). Final yields are a consequence of environmental-dependent losses such as biotic and abiotic stresses (Temesgen et al., 2015; Lyu et al., 2021), adequate plant symbiosis with *Rhizobium*, as well as on the stable wild bees’ population to ensure both optimal seed set and outcrossing rates (O’Sullivan and Angra, 2016).Unraveling the potential of the crop requires the availability of the high-performing varieties across environments but large GEI can reduce selection gains and make identifying superior cultivars more difficult. Therefore, identifying GEI patterns is essential to determine whether a genotype has a wide or a specific adaptation and for creating high-yielding and adaptable cultivars (Gela et al., 2023).

To identify the best faba bean accessions for a given environment requires the assessment of their performance and stability in multi-environment trials (MET) across locations and years whose interpretation can be complex. The additive main effects and multiplicative interaction (AMMI) reported by Gauch and Zobel (1996) and the genotype main effect and GEI interaction (GGE biplot) developed by Gabriel (1971) are the main statistical methods used for analyzing plant yield stability (Greveniotis et al., 2023). Both are frequently utilized together to define mega-environments as well as genotypes with best performances in each mega-environment (Taleghani et al., 2023). Unpredictable weather conditions affect the identification of repeatable GEIs which affect the definition of mega-environments. The definition of mega-environments (ME) for a specific crop within a crop growing area positively affects breeding and selection efficiency, maximizes crop production and facilitates selection of superior cultivars (Yan et al., 2023).

This is overcome by repeating the experiment more than one year and selecting an adequate and experimental design. Partially replicated and randomized complete block augmented designs are some of the solutions to separate interaction and residual error (Elias et al., 2016). A main drawback of utilizing augmented designs is that it may result in inaccurate genotype estimate and ranking, as these rely on the check genotypes (Moehring et al., 2014). On the other hand, with a limited amount of seed, these designs allow the use of a maximum of environments in multi-environmental trials (METs). The GGE biplot is considered to be more effective than the AMMI analysis since it eliminates the statistical effect of the environment and concentrates on genotype evaluation and traits stability (Yan et al., 2007). Besides the above mentioned methods, best linear unbiased predictions (BLUP), has been used to analyze mega-environments and to estimate the mean yield of genotypes and estimating genotypic values in mixed models (Smith et al., 2005; Nardino et al., 2016). Since the introduction of GGE biplot numerous applications of the method have been reported to evaluate GEI and yield stability in several legume crops, such as common bean (Zanella et al., 2019; Souza et al., 2023), cowpea (Haisirikul et al., 2020), winged bean (Akinyosoye et al., 2023) or faba bean (Gurmu et al., 2012; Koç et al., 2018; Greveniotis et al., 2023). However, there is little information on using the GGE biplot method to examine the GxE and stability of traits in large faba bean collections.

In addition to the genotype stability there is a demand for cultivars that perform well in different environments (Memon et al., 2023). Thus, the ideal genotype will be the one that achieves the optimal level of each trait, balancing the negative effect of trait correlations and demonstrating suitable yield across different environments. This prerequisite can be met by the multi-trait stability index (MTSI) recently proposed by Olivoto et al. (2019). This tool is highly efficient in selecting stable genotypes in multi-environment experiments based on multiple traits. Considering the correlation structure among traits, the index implements a selection process for multiple traits that has proven to be useful in different breeding programs (Hassani et al., 2023). Superior, regionally adapted genotypes require fewer resources and lower inputs to maximize production, which is in alignment with the global needs of sustainability.

The objective of this study was to perform a multi-environment and multi-year trial using the GGE biplot and the MTSI methods to evaluate the stability and adaptability of 220 faba bean accessions in order to identify superior genotypes based on key traits related to phenology, morphology and yield components. Field assessment was performed over three years in four representative European agroecological areas to: а) evaluate the diversity and stability of these traits in a worldwide faba bean collection and, b) to identify high yielding and stable genotypes suitable for each agroecological environment across Europe.

## 2 Materials and methods

### 2.1 Faba bean diversity panel

**Figure 1:**
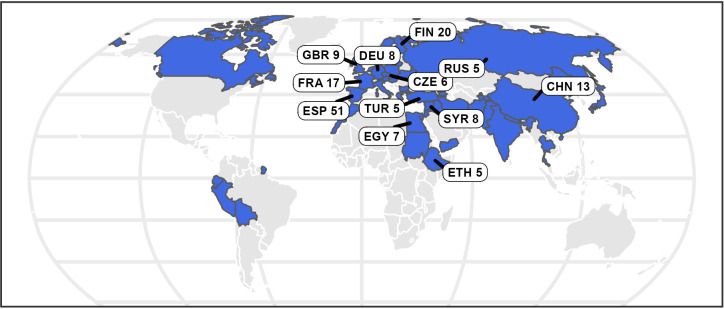
The map displays in blue the origin of the 220 faba bean accessions. The countries providing more than five accessions are indicated with labels.

A collection of 220 faba bean accessions defined within the Eucleg project was selected for the study. Accessions are conserved in five institutions: Centro Nacional de Recursos Fitogenéticos (ESP004); IFAPA (ESP046); UMR1347 Agroecology, Plant Biology and Breeding, INRAE Dijon (FRA043) and Nordic Genetic Resource Center (SWE054), International Center for Agricultural Research in the Dry Areas ICARDA (SYR002). The accessions were categorized in the four basic botanical types according to seed characteristics: major, minor, equina and paucijuga as well as in four transitional forms: equina-major, equina-minor, major-equina and minor-paucijuga. The 220 accessions originated from 42 countries. (**Figure 1**; **Supplementary Table S1**).

### 2.2 Experiment locations and designs

**Figure 2:**
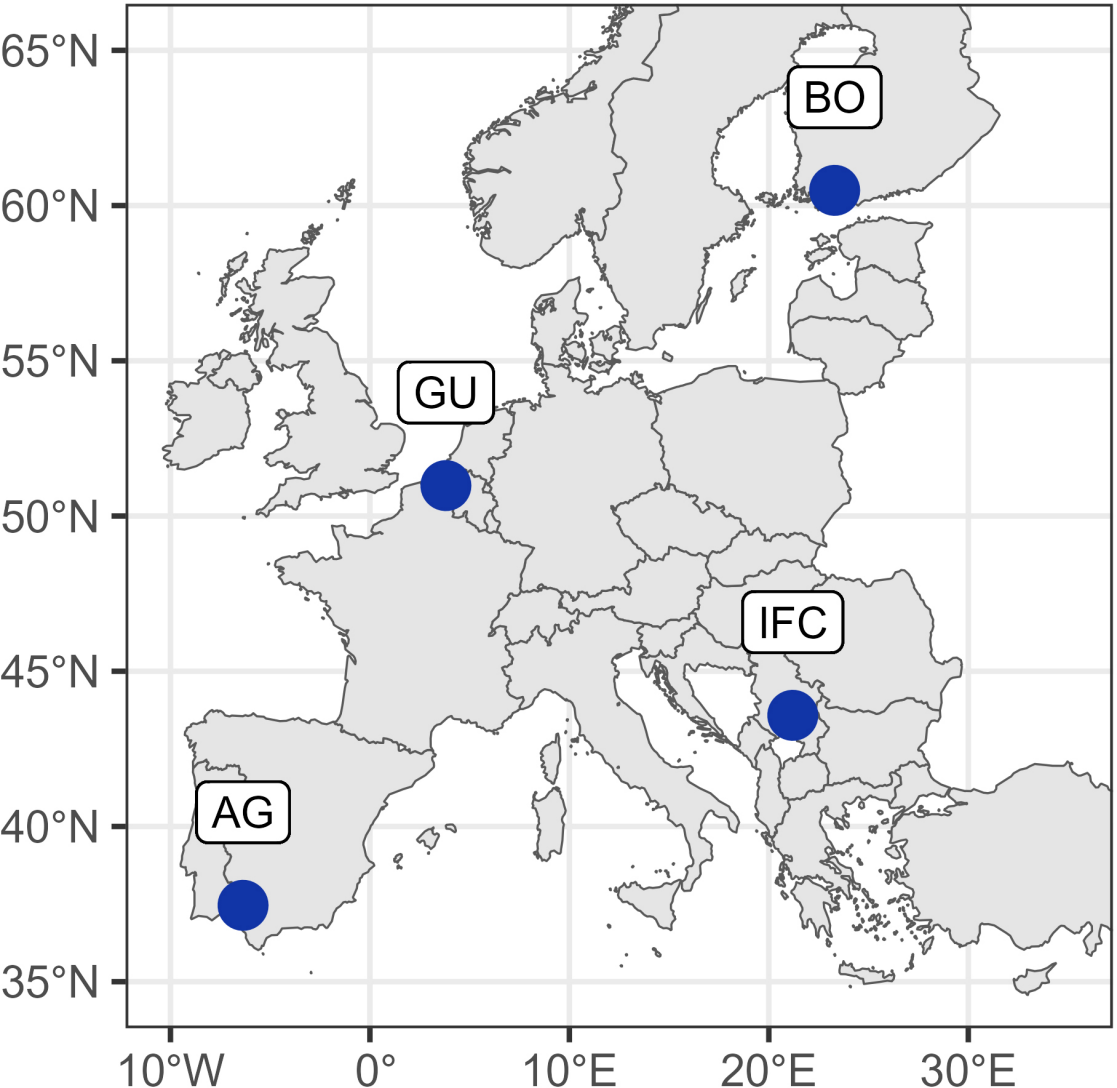
Experiment locations indicated by blue circles: AG (Agrovegetal) – Spain; IFC (Institute for forage crops Kruševac) – Serbia; GU (Gent University) – Belgium; BO (Boreal) – Finland.

The maps in **Figures 1**, **2** were generated using packages rnaturalearth (v0.3.2 Massicotte and South, 2023), Eurostat (v.3.8.2. Lahti et al., 2017).

The experiment was conducted in three years (2018 - 2020) in the network of four field testing sites, covering 4 representative European biogeographic regions: continental, Mediterranean, Atlantic and boreal (https://www.eea.europa.eu/data-and-maps/figures/biogeographical-regions-in-urope-1)^2^. These four fields were located in Spain, Serbia, Belgium and Finland (**Table 1**).

**Table 1:** Information on the partners and acronyms, experimental locations and countries, GPS positions, time frame and soil characteristics.

Partner	Trial Location	Country	Latitude	Longitude	Altitude (m asl)	Type of soil
Agrovegetal (AG)	Escacena del Campo	Spain	37°27’18.3”N	37°27’18.3”N	67	Heavy clay (45% clay, 30% silt, 25% sand)
Boreal (BO)	Jokioinen	Finland	6°21’47.9”W	6°21’47.9”W	39	Clay (55% clay, 10% silt, 35% sand)
Gent University (GU)	Melle	Belgium	60°49’16.4”N	60°49’16.4”N	11	Loamy sand (80% sand, 12% silt, 8% clay)
Institute for forage crops Kruševac (IFK)	Globoder, Kruševac	Serbia	23°29’58.1”E	23°29’58.1”E	149	Clay loam (32% clay, 29% silt, 39% sand)

**Figure 3:**
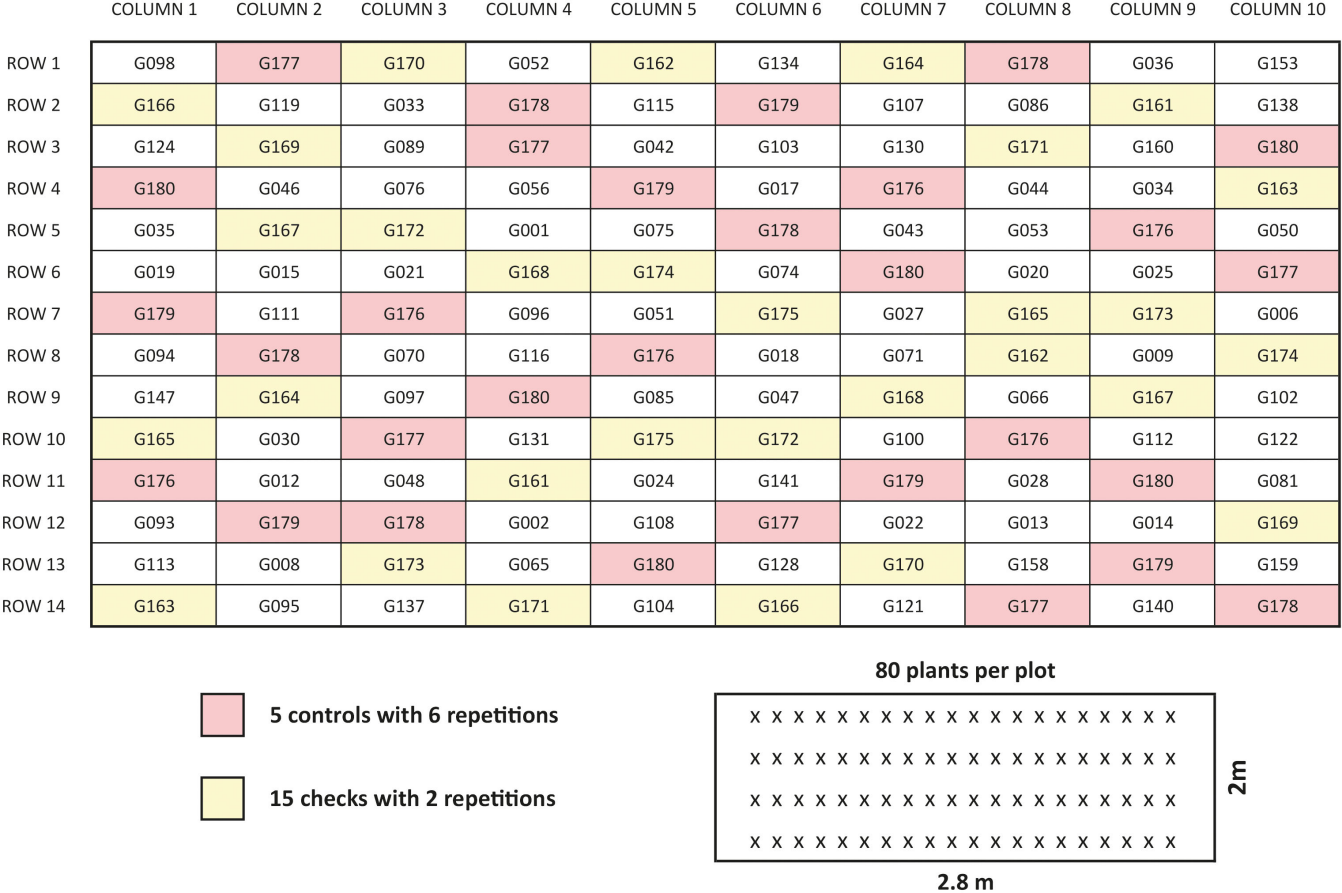
Example of trail design at one location in Serbia (Environments IFC18, IFC19, IFC20). Figure present design 10 columns x 14 rows. One hundred genotypes were distributed in 140 plots, 80 genotypes (white color plots) were repeated once, 5 checks (pink color) were repeated 6 times and 15 checks (yellow color) were repeated two times. Small picture is one single plot with 80 plants distributed evenly in 4 rows.

Nine assays were conducted, three in Serbia during 2018, 2019 and 2020 and two in Spain, Belgium and Finland during 2018 and 2019. Each combination of location and year was treated as a unique single environment and named with the acronym of the center involved and the year of the evaluation (in Serbia: IFC18, IFC19 and IFC20, in Spain: AG18 and AG19, in Belgium: GU18 and GU19 and in Finland: BO18 and BO19) (**Table 2**). In all nine trails 220 genotypes (entries) were tested (**Supplementary Table S1**). Each single trail described as environment with specific acronym, had 100 genotypes set-up using augmented p-rep experimental design (Cullis et al., 2006) in row by column arrangement (14 x 10) and 140 plots. And each trail had 15 checks that were repeated 2 times, 5 standards (cultivars - Mistral, Merkur, Merlin, Fanfare and Baraca) were repeated 6 times and other 80 entries one time. The checks were chosen according to their adaptation to some of the agroecological areas where the assays were performed. Each plot consisted of 4 rows with 0.5-0.7 row spacing and 77-80 seeds (**Table 2**). The example of one field trail layout is illustrated in **Figure 3**. Testing early generations with a limited amount of seeds across multiple locations (four in our experiment) is one of the main advantages of using an augmented p-rep design. Since not all the entries are repeated equally, they are not compared with the same precision. Nevertheless, check plots enable the estimation of block effects, error variances and a connection of otherwise unconnected multi-environment trials (Moehring et al., 2014).

**Table 2:** Single trails description; environmental acronyms begin with two or three letters identifying the trail location (AG- Agrovegetal Spain, IFC Institute for forage crops Kruševac Serbia, BO – Boreal Finland, and GU –Ghent Belgium) followed by the year.

Environments	AG18	AG19	IFC18	IFC19	IFC20	BO18	BO19	GU18	GU19
Number of accessions									
1 plot	80	80	79	79	79	80	78	80	77
2 plots	15	15	15	15	15	15	15	15	15
6 plots	5	5	5	5	5	5	5	5	5
In total	100	100	99	99	99	100	98	100	97
Number of plots	140	140	139	139	139	140	138	140	137
Rows x Columns	14x10	14x10	14x10	14x10	14x10	14x10	14x10	14x10	14x10
Plot size	2m x 2.1m	2m x 2.1m	2m x 2.8m	2m x 2.8m	2m x 2.8m	2m x 2m	2m x 2m	2m x 2m	2m x 2m
Number of rows per plot	4	4	4	4	4	3	3	4	4
Distance between rows within plots	0.7 m	0.7 m	0.7m	0.7m	0.7m	0.7m	0.7m	0.5m	0.5m
Distance between rows between plots	1.5 m	1.5 m	0.7 m	0.7 m	0.7 m	0.7m	0.7m	0.5m	0.5m
Sowing depth (in cm)	3 cm	3 cm	3 cm	3 cm	3 cm	6 cm	6 cm	5 cm	5 cm
Sowing density (seeds m2)	20	20	14	14	14	12	12	14	14
Sowing date	2018-11-16	2019-11-16	2018-04-25	2019-03-27	2020-03-03	2018-06-02	2019-06-02	2018-0418	2019-04-04

The agronomic treatments (fertilizers, insecticides, and herbicides) were applied following common local practices. Trials were sown in spring and harvested in summer, except in southern Spain, where the growing season was from November to June (**Supplementary Tables S2A, B**).

### 2.3 Phenotyping

Plants were measured for nine traits related to: phenology - days to full flowering (DTF, recorded as the date when 50% of plant have flowered) and days to full maturity (DTM, date when of pods have ripened); morphology - plant height (PH, measured near maturity, from the ground to the top of the plant, in cm), plant branching (NOB, number of branches from basal nodes), height at which the first pod appears (HFP, in cm); and seed yield components affecting the final yield and therefore relevant for faba bean breeding - number of pods per node (PPN, mean value of five nodes from the main stem), number of pods per flower (PPF, number of pods per node divided by the number of flowers per node), pod length (PL, in cm, measured on 10 pods from the main stem) and number of seed per pod (SPP, mean of seeds from the previous pods) (**Supplementary Tables S3A, B**). All data were collected from plants in the two central rows of the plot and given as average values.

Genotypes were categorized in four main and four transitional botanical types (**Supplementary Table S1**): Paucijuga, Minor-Paucijuga, Minor, Equina-Minor, Equina, Major-Equina, Major, and Equina-Major. In the final analysis, transitional seed types were merged with adequate main types and presented as four groups (**Table 3**).

**Table 3:** Percentage of botanical types (paucijuga, minor, equina and major) in each subcluster and in the whole collection.

Subcluster	A	B	C	D	E	F	G	Whole collection
Paucijuga	12	1	0	0	4	0	7	5
Minor	18	21	0	4	65	17	72	24
Equina	48	48	32	50	19	33	14	41
Major	11	10	47	46	4	17	0	17
Unknown	11	20	21	0	8	33	7	13

### 2.4 Statistical analysis

Data analyses were performed using R software (R Core Team, 2022) and the Progeno^©^ (Progeno, 2020) software framework (https://www.progeno.net). Data were checked for homogeneity of the variances in each environment with box plots, histograms, scatter plots and heat maps.

The standardized BLUP (Best Linear Unbiased Prediction) values of genotype effects for the nine traits, which takes into account known or estimated variances (Liu et al., 2008) were generated by the Progeno^©^ (Progeno, 2020) database and the software package.

**Descriptive statistics:** For each of nine traits, data was summarized using the following descriptive statistics parameters: mean, standard deviation, standard error, coefficient of variation, minimum, maximum, and range. Descriptive parameters were calculated for each environment and each trait separately (**Supplementary Table S4**).

**Estimation of variance:** To evaluate the genotypes stability across the environments, multi-environment trials (METs) data were subjected to a multi-trial linear mixed model analysis in Progeno^©^ (Progeno, 2020). The multi-trial analysis always performs a trial connectivity analysis based on the use of common accessions (checks) within and among trials. The variance parameters were estimated by restricted maximum likelihood (REML) assuming genotype and GEI as random effects. The most commonly used approaches for estimation of variance components in linear mixed models (LMM) are maximum likelihood (ML), restricted maximum likelihood (REML), and minimum norm quadratic unbiased estimation (MINQUE). ML and REML approaches are integrated into R package lme4, which we used to perform LMM. REML is commonly used in linear mixed models in which the variance components of the random effects need to be estimated, while ML is commonly used when the goal is to estimate both fixed and random effects. In this paper, our main goal was to estimate variance components for the genotype, which is a random effect, hence we selected the REML approach. It has to be indicated that under a multi-trial linear mixed model the likelihood has converged and that the variance estimation procedure has finished normally.

**Heritability calculation:** The coefficient of generalized heritability (H^2^) was calculated using the method of Cullis et al., 2006, where the H^2^ is a function of the reliability of the BLUPs (vblup - the average standard error of differences between BLUPs squared), and the genotypic variance (var_g). This method is used in unbalanced dataset where all genotypes are not equally observed in every trial.


$$H^{2}=1-\frac{vblup}{2\;*\;varg}$$

To obtain the BLUPs values we used a linear mixed model fitted by REML using the function ‘lmer’ from ‘lme4’ R package (v1.1.31; Bates et al., 2015) where we considered the variable genotype (G) as random effects and the variable environment (E) as fixed effects. The H^2^ was also calculated for each environment, where we considered the variable genotype (G) as random effects.

**Botanical types:** The effect of the botanical types on the nine response variables was analyzed using a linear mixed effect model with ‘lmer’ function from ‘lme4’ R package (v1.1.31; Bates et al., 2015). We considered the botanical types (paucijuga, minor, equina and major) as fixed effects and Environment (E) and Genotype (G) as random effects. For each response variable the model intercept corresponded to the botanical type paucijuga. The R package ‘lmerTest’ (v3.1.3; Kuznetsova et al., 2017) was used to approximate degrees of freedom and calculate p-values for the fixed effects.

**Correlations:** The Pearson correlation coefficients among traits and environments were estimated using the ‘rcorr’ function of ‘Hmisc’ R package (v5.0.1; Harrell, 2023). Correlations were visualized using the ‘ggcorrplot’ R package (v0.1.4; Kassambara, 2022).

**GGE Biplot model:** The biplot is a popular data visualization tool introduced by Gabriel (1971), that allows to understand the GxE effects and to detect genotypes particularly adapted to specific environments. Observations and variables are represented in a single graph allowing the detection of groups within the observations while also showing the dispersion and correlations between variables or columns (Hongyu et al., 2014; Gauch, 2006). GGE Biplot analysis (Yan et al., 2000) displays both the genotype main effects (G) and the GEI effects from multi-environment trials (MET). Plant breeders have found GGE Biplots very useful in mega-environment analysis (Flores et al., 2013; Rubiales et al., 2014) and genotype evaluation (Araújo et al., 2022; Lal et al., 2022). Although the GGE biplot does not separate genotype effect (G) from the GEI (Araújo et al., 2022) it was concluded that the GGE is equal to or superior to the AMMI proposed by (Gauch, 2006) in three main aspects of genotype by environment data (GED) analysis: mega-environment analysis, genotype evaluation, and test-environment evaluation.

The GGE Biplot is based on decomposing the data matrix Y (which contains g rows representing genotypes and e columns representing environments) by singular value decomposition (SVD) into p principal components with p ≤ (e ,g−1) as:


$$Y_{ij}-\mu_{j}=\lambda_{1}\alpha_{i1}\gamma_{j1}+\;\ldots \;+\lambda_{t}\alpha_{it}\gamma_{jt}+\epsilon_{ij}\;$$

where $Y_{ij}$ is the mean performance of genotype i in environment *j*, $\mu_{j}$ is the mean value of environment *j*, and *t* is the number of principal components. $\left[\alpha_{ij}\right]$ is genotype scores matrix, $\left[g_{ij}\right]$ is the environment scores matrix, $\lambda_{t}$ are singular values. The model constraints are: (i) $\lambda_{1}\geq \lambda_{2}\geq \ldots \geq \lambda_{t}\geq 0$ , (ii) matrices $\left[\alpha_{ij}\right]$ and $\left[g_{ij}\right]$ are orthonormal (Araujo et al., 2022).

The GGE Biplot is constructed using the first two PCs. The genotype coordinates are $\left(\lambda_{1}^{f}\;\alpha_{i1},\;\;\lambda_{2}^{f}\;\alpha_{i2}\;\right)\;$ while the coordinates of environments are $\left(\lambda_{1}^{1-f}\;\alpha_{i1},\;\;\lambda_{2}^{1-f}\;\alpha_{i2}\;\right)$ . The exponent f , with 0≤f≤1 is used to rescale the genotype and environment scores to enhance the visual interpretation of the biplot. “Cultivar focused” scaling has f=1 , the “environment focused” scaling has f=0 and for f=0.5 we have “symmetric scaling” (Yan and Hunt, 2002).

#### 2.4.1 Mega-environment analysis

A mega-environment is a group of locations that constantly share the best set of genotypes across years (Yan and Rajcan, 2002). For the identification of the best genotypes among the faba bean panel, we conducted a mega-environment analysis (“which-won-where” view of the GGE model) and a genotype evaluation (“mean vs. stability” pattern of the GGE model) based on the scores of the nine traits in the nine environments. The mega-environment analysis consists of an irregular polygon and a set of lines drawn from the biplot origin intersecting each of the sides at right angles. The genotypes at the vertices are the ones located farthest from the biplot origin in various directions. The polygon contains the rest of the entries, and the lines starting at the origin and intercepting perpendicularly the polygon side represent hypothetical environments in which two genotypes at the end of the corresponding side perform equally. The lines radiating from the origin divide the biplot into sections and there is a vertex (genotype) for each section that had the best yield performance in the environments contained in that section, which is called a mega-environment (Flores et al., 2013). If the environments are located in different sectors, this means that different genotypes won in different environments, so the original set of environments can be divided into two or more mega – environments.

#### 2.4.2 Genotype evaluation

The genotype evaluation was performed for each mega-environment separately using the Average Environment Coordination (AEC) view of the GGE Biplot (Yan, 2001), also called “Mean vs. Stability” view. The abscissa, or average environment axes (AEA), passes through a biplot origin and an average environment point, which is located at the mean of PCA1 and PCA2 scores. It has a single arrow that is pointing toward a greater mean performance for a selected trial. The genotype performance is ranged according to its projection on the AEA. The average environment axes represent the main effect of a genotype (G contribution to the G + GE model).

The second (ordinate) axis passes through the origin and is perpendicular to the abscissa. The ordinate represents the stability of genotypes across all environments. Stable genotypes tend to have smaller projection lengths on the ordinate, so they are closer to the average environment axes. The ideal genotype has the highest performance and it is absolutely stable as it is located on the AEC abscissa (Yan and Kang, 2003). All other genotypes are ranked based on their distance from the ideal genotype.

Genotype as main effect and GEI were analyzed and visualized by GGE biplots for all investigated traits separately as described by Yan and Kang (2003). Results are presented in “which won where” biplots (Yan et al., 2007), an effective graphic tool in mega-environment analysis and by the Average environment coordination (AEC) GGE view (Yan et al., 2000) that analyzes both performance and stability within each mega-environment.

**MTSI:** The multi-trait stability index (MTSI) was calculated for phenotypic (PH, NOB, HFP) and seed yield-related traits (PPF, PL, PPN, SPP). For PH, PPF, and PL, where only one mega-environment was detected, all environments were included into the analysis. For the remaining traits (NOB, HFP, PPN, and SPP), we performed two multi-trait stability analyses, one for each of two mega environments detected.

Prior to the MTSI evaluation, we defined the ideotype. The ideotype values for PPF and SPP were maximum values obtained for these two traits, 1 and 8.33, respectively. For the other traits, the ideotypes were established as follows: PH = 93 cm, PL = 9 cm, NOB = 4.9, HFP = 10 cm, and PPN = 4.12.

For each trait X, phenotypic data were transformed using the following formula:


$$Y_{i}=\{\begin{array}{c} I+\left|X_{i}-I\right|\;:\;\;I<\frac{X_{min}+X_{max}}{2}\\ I-\left|X_{i}-I\right|\;:\;I\geq \frac{X_{min}+X_{max}}{2}\; \end{array}\;\;\;\;$$

Where I is the ideotype value for a selected trait, $X_{min}$ and $X_{max}$ are the minimum and maximum values for the trait, respectively, and $X_{i}$ is the original phenotypic value. After this transformation, the most desired value is either the largest or smallest for each trait; furthermore, all $Y_{i}$ values are greater than zero.

The simultaneous selection for mean performance and stability was performed by using the WAASBY index, which allows weighting between stability (WASSB) and performance (Y). Y is the matrix of the response variables, where rows are genotypes and columns are traits. WAASB is the weighted average of absolute scores from the singular value decomposition (SVD) of the best linear unbiased prediction matrix for the GE interaction effect. To compute the WAASB index, we used the function waasb() of the metan R package (Olivoto and Lúcio, 2020).

The WASSBY index for i-th genotype was calculated as:


$$WAASBY_{i}=\frac{rY_{i}\;*\;\Theta_{Y}+rW_{i}\;*\;\Theta_{S}}{\Theta_{Y}+\Theta_{S}}$$

where $\Theta_{Y}$ and $\Theta_{S}$ are the weights for the response variable and the WAASB index, respectively. In this paper, we added a higher weight for mean performance ( $\Theta_{Y}$ = 70) than for stability ( $\Theta_{S}$ = 30). This weighting prioritized performance over stability to avoid selecting highly stable genotypes with low performance. $rY_{i}$ and rWi are rescaled values for the response variable and WAASB, respectively.


$$rY_{i}=rW_{i}=\frac{a-b}{Omax-Omin}\left(O_{i}-Omax\right)+a$$

$O_{i}$ is the original value for response variable (or WAASB) for i-th genotype. $O_{min}$ and $O_{max}$ are minimum and maximum values for the original variable. The values a  and b were chosen such that a=100 and b=0 , when the highest values are desirable and when the lowest values are desirable, a=0 and b=100 . After rescaling, in both, response and WAASB matrices, the columns will range from 0 to 100. The best genotypes will have a score of 100, while in the worst ones the score is 0. Hence, the most stable genotypes have quite the same values as ideotype (100), while the most unstable have a score of 0. The ideotype has a score of 100 for each trait.

The MTSI is computed based on factor analysis considering a selection intensity of 15%. The genotype ranking is based on Euclidean distance between genotype and ideotype. The formula for evaluating MTSI for i-th genotype is:


$$MTSI_{i}=\sqrt{\sum_{j=1}^{f}{\left(F_{ij}-F_{j}\right)}^{2}}$$

where MTSI is the multi-trait stability index for the i-th genotype, F is the ( g x f ) matrix with the factorial scores ( g is the number of genotypes and f is the number of retained factors), $F_{ij}$ is the jth factorial score of the ith genotype, and Fj is the jth score of the ideotype. Factorial scores were calculated based on factor analysis for genotypes and traits using the WAASBY matrix, matrix of canonical loadings and correlation matrix between traits.

The genotypes with low MTSI score are more similar to the ideotype and hence have a high mean performance and stability for all traits under study. To compute the multi-trait stability index, we used the function mtsi() of the metan R package (Olivoto and Lúcio, 2020).

**Clustering:** Best linear unbiased predictors (BLUP) obtained for all the traits are used to perform Hierarchical Cluster Analysis. The genotypic distance matrix, based on a scaled matrix of BLUP-s with 220 rows (genotypes) and 9 columns (trials), was constructed using Euclidean distance measure. The phenotypic distance matrix for the nine traits was constructed based on the Pearson correlation coefficient. We used Euclidean distance to measure average cluster diameter and the complete linkage method to measure distance between clusters. Two dendrograms, based on the genotypic and phenotypic distance matrix, were constructed using the complete linkage method with the ‘dendextend’ R package (v1.17.1; Galili, 2015). Hierarchical Cluster Analysis Heatmaps were created using the ‘heatmap.2’ function from ‘gplots’ R package (v3.1.3; Warnes et al., 2022). Average interclass and intraclass distances were computed using the ‘clv’ R package (v0.3.2.2; Nieweglowski, 2020).

**PCA:** We performed principal component analysis (PCA) based on BLUPS to visualize the relationships between the nine phenotypic traits and the 220 genotypes. Also, the PCA was used to further examine the results obtained by the Hierarchical Clustering Method in section 3.6. In order to visualize differences between the four botanical types, we conducted a PCA analysis based on standardized BLUPS of those genotypes for which the botanical type was known. All PCA analyses were done using the ‘prcom’ function from ‘Stats’ R Package.

## 3 Results

### 3.1 Descriptive statistical analysis of phenotypic traits

All trails, except in Spain, were established in the spring of 2018, 2019, and 2020. Because of the different climatic conditions in Spain (environments AG18 and AG19), sowing occurred in the autumn and the trials were completed in the spring (June) of the following year. The number of days to full flowering (DTF) at the different environments ranged from 28 days (IFC20) to 108 days (AG18) with a mean of 80 days, while full maturity (DTM) varied by almost 116 days between the earliest genotypes (68 days in IFC19) and the latest genotypes (184 days in AG18). As a result of the autumn sowing, Spain required a longer average time than other environments for full flowering (80.2 - 90.4 days) and ripening (156 - 166 days). The Serbian location in season 2019 (IFC19), was the earliest environment and all accessions mature after an average of 78 days (**Supplementary Table S3B**).

The CV (coefficient of variation) of days to full flowering (DTF) exceeded 13% in the five environments (max values 18.1 in BO19), whereas the day to mature variability was obviously higher across environments (CVs below 10%).

The maximum plant height (PH) value (154 cm) was recorded in environment AG19 which also showed the highest average values for this trait (93.3 cm). The shortest plants were detected in BO19, reaching only 34.4 cm in average due to unfavorable weather conditions during the growing season. The variability of this trait was high in all environments exceeding 17.4% for coefficient of variation. The average value of the number of branches (NOB) was very low in GU18 (1.78) and BO19 (2.02). Although branching was quite low in BO19, this environment showed the highest variability (CV close to 60%). Single plants in Spain (AG18) even reached to develop 13 branches and revealed the highest mean value (4.9). The height of the first pod (HFP) showed the highest mean and maximum value in AG18 (34.4 cm and 79 cm, respectively). BO19 recorded the lowest value of HFP (4 cm) and a mean of 16.1 cm. The number of pods per node (PPN), measured on the central part of the main stems, was quite low. Mean values in each environment ranged from 0.47 (BO19 and highest CV of 55.1), to 1.92 (IFC19). Concerning the number of pods per flower (PPF), the lowest mean values were in Spain (AG18, AG19) and BO19. The most productive environments were BO18 with a maximum of 0.85 and IFC18 with 0.94 pods per flower. For some accessions in GU19 almost every flower in the central nodes developed a pod (1.0). Pod length (PL) mean values ranged between 5.39 cm (BO18) and 9.0 cm (AG19). The longest pods were developed in Spain, measuring 18.4 cm (AG18) and 17.7 cm (AG19). Finally, the number of seeds per pod (SPP) ranged between 2.27 and 3.71 in different environments. Some accessions in AG19 and IFC20 showed more than eight seeds per pod in average and very long pods, more than twice longer than the average pod length for all environments (6.83) (**Supplementary Table S4**). Seeds per pod (SSP) and pod length (PL) are traits regularly highly correlated, since longer pods have more seed beds for possible seed development than the shorter ones.

### 3.2 Estimation of variance components

The mixed model analysis of the nine trials in different environments revealed full trial connectivity for all the traits. The REML ANOVA for estimating the variance parameters indicated that the likelihood converged, meaning that the estimation procedure finished normally and that the resulting variance components effectively maximize the likelihood function. The estimation of variance components with multi-trial mixed models revealed that the variances of the genotypes or their interactions with the environments in most of the traits were higher than the variances originating from unidentifiable effects (residuals) (**Table 4**). The highest genotypic variances percentages were detected for DTF (42.3%), PH (54.3%), HFP (45.9%) and PL (61.8%). GxE interaction variances were very high for all traits and especially for DTM (47.0%), NOB (34.2%), PPN (46.6%), PPF (44.9%) and SPP (31.2%) while row and column variances were quite low (lower than 5.6%). SPP was the only trait in which the residual variance was higher than both the genotypic and the GxE variance. We calculated broad sense heritability for all nine traits (**Table 4**) and independently in each of the nine environments (**Supplementary Table S5**). The trait heritability exceeded 0.7, with the highest values being 0.914 for plant height and 0.932 for pod length, which correspond to genotypic variance values. The influence of environment on heritability reached values above 0.5 and the Serbian environments were the highest.

**Table 4:** Variance and broad sense heritability estimation of multi-environment trial data by Multi-trial linear mixed model analysis of variance.

	Variances	Genotypic variance		Genotype x environment variance		Row and column variance		Residual variance		Heritability in broad sense
	Traits		%		%		%		%	
1	DTF	16.7	42.3	9.91	25.1	2.19	5.6	10.6	27.0	0.876
2	DTM	12.3	28.4	20.4	47.0	2.17	5.0	8.49	19.6	0.809
3	PH	127	54.3	56.2	23.9	6.07	2.60	45.1	19.2	0.914
4	NOB	0.60	33.1	0.62	34.2	0.03	1.80	0.56	30.9	0.844
5	HFP	33.9	45.9	13.8	18.6	2.02	2.70	24.2	32.7	0.882
6	PPN	0.04	19.2	0.01	46.6	0.01	2.90	0.06	31.3	0.744
7	PPF	0.01	27.4	0.01	44.9	0.00	0.32	0.01	27.4	0.813
8	PL	1.91	61.8	0.57	17.6	0.03	0.9	0.64	19.8	0.932
9	SPP	0.12	28.1	0.13	31.2	0.01	0.6	0.16	40.2	0.813

DTF, days to flower; DTM, days to mature; PH, plant height; NOB, number of branches per plant; HFP, height of first pod; PPF, number of pods per flower; PPN, number of pods per node; PL, pod length; SPP, number of seeds per pod.

### 3.3 Botanical types

The results of the linear mixed model for each trait are shown in **Table 5**. The intercept in the model corresponds to the botanical type paucijuga and the results explain the effect of the botanical type on the nine traits.

**Table 5:** Results from the linear mixed model showing the effects of the botanical type (minor, equina and major) on the nine evaluated traits.

	DTF	DTM	PH	NOB	HFP	PPF	PPN	PL	SPP
Intercept	54.4**	108**	71.28**	2.76**	24.1**	0.35**	1.65**	5.01**	3.23**
Minor	1.56	4.17*	5.45	0.23*	5.61*	-0.070	-0.07	1.03**	-0.08
Equina	-0.97	1.14	-5.96	1.05**	1.65	0.027	-0.29**	1.83**	-0.37**
Major	-0.89	1.63	-8.36*	1.31**	1.24	0.015	-0.43**	3.21**	-0.36*

DTF, days to flower; DTM, days to mature; PH, plant height; NOB, number of branches per plant; HFP, height of first pod; PPF, number of pods per flower; PPN, number of pods per node; PL, pod length; SPP, number of seeds per pod. *p< 0.05, **p< 0.01

The traits most significantly affected by the botanical type were number of branches (NOB), pod length (PL), pods per node (PPN) and number of seeds per pod (SPP) (**Table 5**). In the case of days to mature (DTM) and height of first pod (HFP) the effect of the botanical type minor was statistically significant and positive in comparison with paucijuga. For plant height (PH), the major type was statistically significant and negative, meaning that in all environments genotypes with major seeds produced shorter plants compared with the paucijuga type. The effect of the fixed factor, botanical type, had no significant effect on DTF and PPF.

The seven traits affected by the botanical type are presented in boxplots (**Supplementary Figure S1**). Minor types (green boxplots) for days to mature (DTM) were in the higher positions suggesting that these genotypes took a longer period to mature in all environments. A similar boxplot display was observed for height of the first pod (HFP) and plant height (PH). Paucijuga types in IFC18 needed a similar period for ripening and developed the first pod higher on average. Compared to paucijuga, all the botanical types revealed statistically significant positive effects on the number of branches (NOB) and pod length (PL) while the effect of botanical types were negative for pods per node (PPN) and number of seeds per pod (SPP).

### 3.4 Correlations

**Figure 4:**
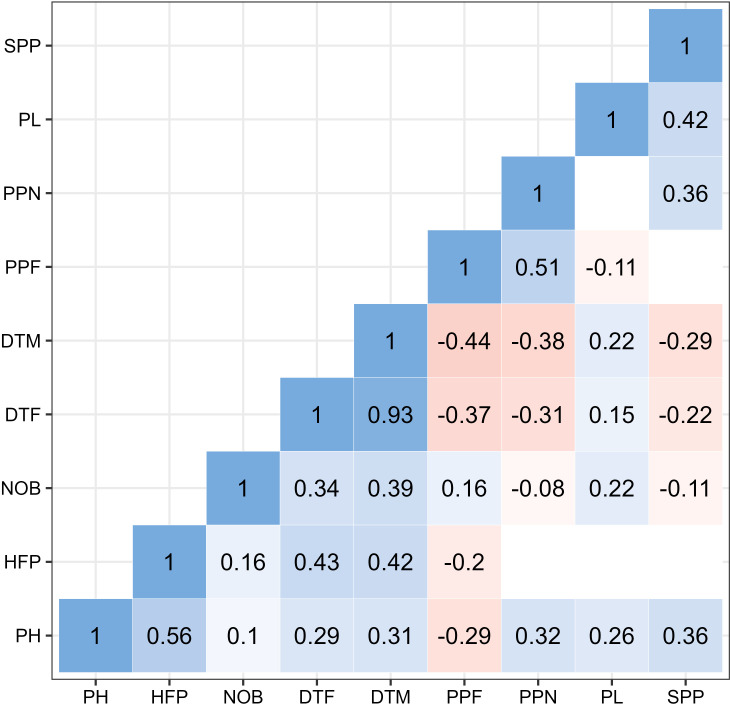
Correlation between nine traits (PH, Plant height; HFP, Height of first pod; NOB, Number of branches; DTF, Days to flower; DTM, Days to mature; PPF, Pods per flower; PPN, Pods per node; SPP, Seeds per pod). The values represent statistically significant correlations at p< 0.01. Blank spaces represent insignificant correlations.

As shown in **Figure 4**, most of the evaluated traits showed positive correlations among them (blue color) and only five of them were not statistically significant. Strongest correlations were observed between days to flowering (DTF) and days to mature (DTM) (r = 0.93, p<0.01), followed by correlation between plant height (PH) and height of the first pod (HFP) (r = 0.56, p< 0.01). Plant height (PH) was positively correlated with all traits except with the number of pods per flower (PPF) (r = -0.29, p<0.01). Height of the first pod (HFP) was moderately correlated with phenotypic traits for DTF (r = 0.43) and DTM (r = 0.42). The highest positive correlation of number of branches per plant (NOB) was with DTM (r = 0.39). Phenological traits (DTM and DTF) were moderately negatively correlated with number of pods per node (PPN) and per flower (PPF) as well as with number of seeds per pod (SPP). Positive correlation was also observed for pod length (PL) and number of seeds per node (SPP) (r = 0.43).

Days to mature (DTM) along with days to flower (DTF) were negatively correlated with pods per flower (PPF) and pods per node (PPN) respectively, where r ranged from -0.44 to -0.31. The remaining negative correlations were lower or negligible (-0.3< r< 0).

**Figure 5:**
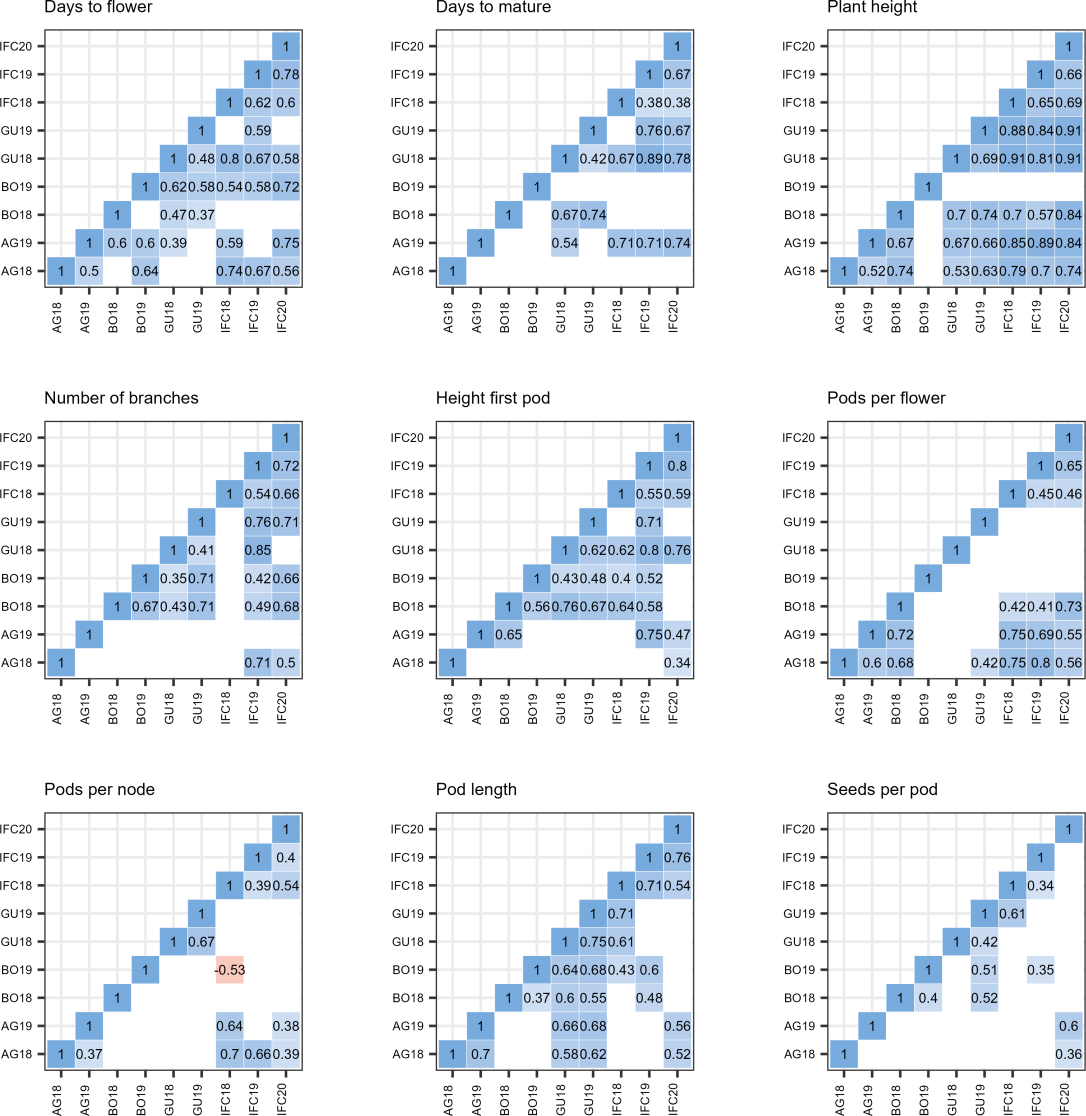
Pearson pairwise correlation coefficient between nine environments in nine trials. environmental acronyms begin with two or three letters identifying the trail location (AG, Agrovegetal Spain; IFC, Institute for forage crops Kruševac Serbia; BO, Boreal Finland; and GU,Ghent Belgium) followed by the year. PH, Plant height; HFP, Height of first pod; NOB, Number of branches; DTF, Days to flower; DTM, Days to mature; PPF, Pods per flower; PPN, Pods per node; SPP, Seeds per pod. Only significant correlations are displayed (p< 0.01).

We also conducted pairwise correlation analyses among environments for each trait (**Figure 5**). All statistically significant correlations were positive except between BO19 and IFC18 for PPN where we observed a moderate negative correlation (r = -0.53, p< 0.01).

### 3.5 GGE Biplot

#### 3.5.1 Which - won - where pattern

**Figure 6:**
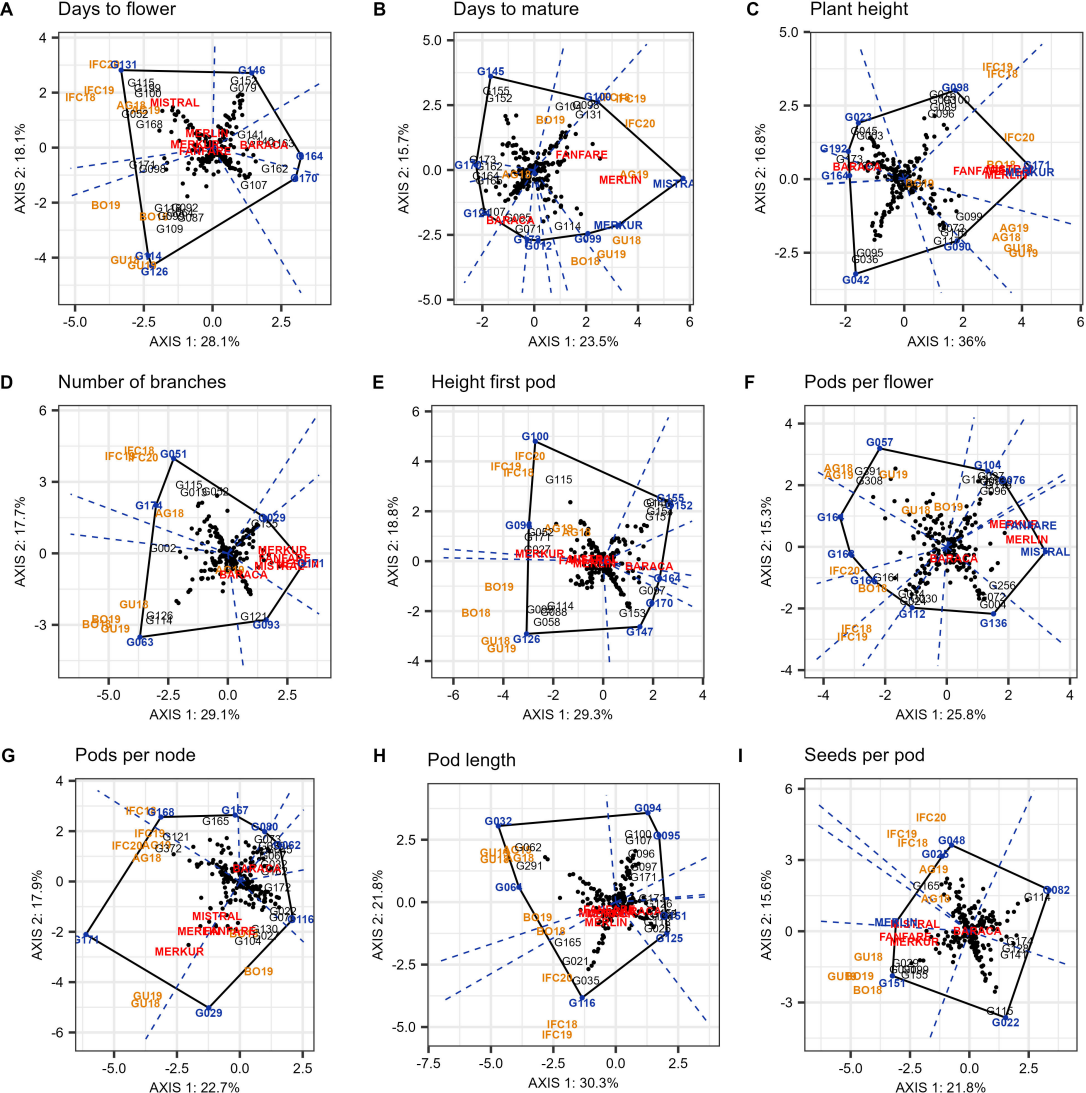
“Which-won-where” pattern of GGE biplot polygon view displaying the G + GE effect for days to flower **(A)**, days to mature **(B)**, plant height **(C)**, number of branches **(D)**, height first pod **(E)**, pods per flower **(F)**, pods per node **(G)**, pod length **(H)**, and seeds per pod **(I)** of 220 faba bean genotypes in nine environments (environmental acronyms begin with two or three letters identifying the trail location (AG- Agrovegetal Spain, IFC Institute for forage crops Kruševac Serbia, BO – Boreal Finland, and GU –Ghent Belgium) followed by the year). The biplots were based on centering = 0, SVP = 2, and scaling = 0.

The which-won-where pattern of the GGE biplot polygon view based on 220 genotypes and nine environments for the nine evaluated traits is shown in **Figure 6** and as a **Supplementary Figures S2A–I**. The first two principal components (PC1 and PC2) of the GGE Biplot explained 46.2%, 39.2%, 52.8%, 46.8%, 48.1%, 41.1%, 40.6%, 52.1% and 37.4% of the variation for DTF, DTM, PH, NOB, HFP, PPF, PPN, PL and SPP, respectively.

The polygon view revealed which group of locations consistently shared the best set of genotypes across years. Therefore it was determined that for days to flower (DTF), number of branches (NOB), height of the first pod (HFP), pods per node (PPN) and seeds per pod (SPP) all nine environments can be divided into two different mega-environments. The first included the three assays from Serbia (IFC18, IFC19 and IFC20) and the two from Spain (AG18 and AG19) and may be referred to as the South European Mega Environment (SE-ME). On the other hand, the two environments from Belgium (GU18 and GU19) and the two from Finland (BO18 and BO19) were grouped into a second mega-environment, which can be referred to as the North European Mega-Environment (NE-ME) (**Figure 6**).

Days to flower (DTF) was divided into six sectors delimited by the perpendicular lines to each side of the polygon (**Figure 6A**), and two sectors or mega-environments. In the first mega-environment (SE-ME) the best genotype was G131, followed by G115, G199, G100 and cultivars Mistral, while in the second mega-environment (NE-ME) the winning genotype was G126. Between these two sectors, G114 located in the vertex of a small sector was the highest yielding line in both mega-environments. For the number of branches (NOB) (**Figure 6D**), the winning genotypes were G051 and G063 in SE-ME and NE-ME, respectively. The biplot was divided into six sectors, among which the two larger ones were representing mega-environments. The genotype with lower number of branches in both environments was G171 as well as four of the checks used in the augmented p-rep design (Merlin, Fanfare, Mistral and Merkur). The biplot for height of the first pod (HFP) identified eight sectors. The highest yielding genotype in SE-ME was G100 followed by G115. In NE-ME the best genotype was G126, along with G058, G099, G088 and G114 (**Figure 6E**). Genotype G098 showed the greater height of the first pod in both mega-environments. Concerning pods per node (PPN), the highest number in the SE-ME was detected in G171, while G029 was the best in the NE-ME (**Figure 6G**). Standard cultivars like Merkur also showed a high number of pods per node in all environments. The best genotype for seeds per pod (SPP) in SE-ME was G048, while the winner for the second mega-environment was G151 (**Figure 6I**).

Plant height (PH), pods per flower (PPF), pod length (PL) and days to mature (DTM) did not show repeatability over the years, so the target environment could not be divided into two or more mega-environments. In the case of PH (**Figure 6C**), environments from Spain and Belgium (AG and GU) were in the same sector and the best performer was the cultivar Merkur. The environments from Serbia and especially Finland (IFC and BO) were mixed up and did not show a clear year-to-year pattern. Winning genotypes in these environments were G171 and G098. For PPF (**Figure 6F**), we detected 10 sectors, four of which contained one or more environments. G057 was the best in Spain, Belgium and in Finland 2019. In case of PL (**Figure 6H**), G116 was the winner all the years in Serbia, while the best for the rest of the locations were G032 and G064. Finally, for DTM (**Figure 6B**) nine different sectors were detected and the winning genotype was the check Mistral in all environments except in BO18, BO19 and AG18 which were distributed in different sectors.

#### 3.5.2 Mean vs. stability pattern

For each mega-environment, we constructed the ‘mean vs. stability’ (Average Environment Coordinate (AEC) view of a GGE model that helps to simplify the genotype assessment based on the mean performance and stability under each mega-environment. The AEC abscissa is the line that passes through the average environment and the biplot origin with an arrow that ranks the genotypes in increasing order with respect to mean performance. The AEC ordinate or stability axis, is a line perpendicular to the X-axis that passes through the plot origin. The effect of the environment increases with the distance from the ordinate axis indicating lower stability in both directions.

**Figure 7:**
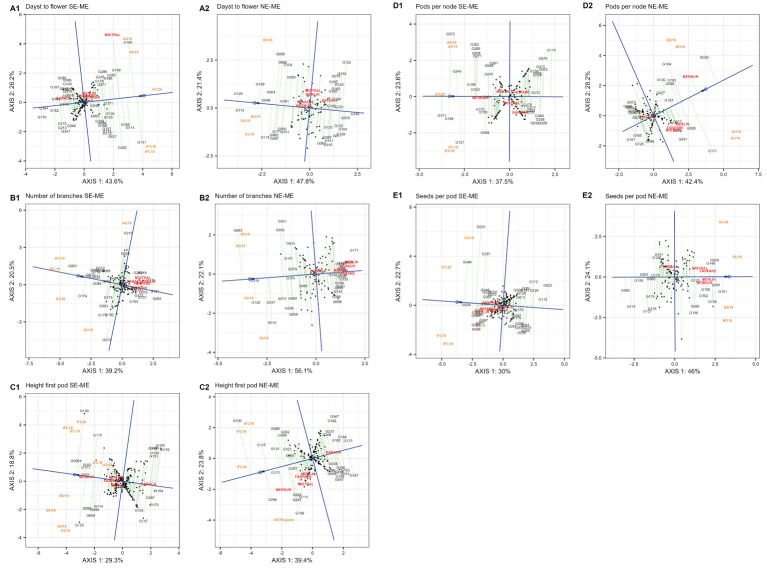
**(A)** “Mean vs. stability” pattern in two mega-environments of 220 faba bean genotypes for days to flower (A1, A2), number of branches (B1, B2) and height first pod (C1, C2). The biplots were based on centering = 0, SVP = 2, and scaling = 0. 7 **(B)** “Mean vs. stability” pattern in two mega-environments based on 220 faba bean genotypes for pods per node (D1, D2) and seeds per pod (E1, E2). The biplots were based on centering = 0, SVP = 2 and scaling = 0.

The results obtained for the ‘mean vs. stability’ pattern of GGE biplot revealed 69.8% (SE-ME) and 69.2% (NE-ME) for days to flower (DTF) (**Figures 7A**, A1, A2); 59.7% (SE-ME) and 78.2% (NE-ME) for number of branches (NOB) (**Figures 7A**, B1, B2); 63.2% (SE-ME) and 80.6% (NE-ME) for height first pod (HFP) (**Figures 7A**, C1, C2); 61.1% (SE-ME) and 70.6% (NE-ME) for pods per node (PPN) (**Figures 7B**, D1, D2) and 52.7% (SE-ME) and 70.1% (NE-ME) for seeds per pod (SPP) of G + G x E variation, respectively.

The higher number of days to flower in SE-ME was detected in G131, followed by G199, G115, G100, G168 and Mistral, but these genotypes showed low stability due to their far position from the AEC abscissa line. Genotypes G170, G164 and G162 were the ones more stable and early flowering in SE-ME. In the mega-environment NE-ME, the latest flowering genotypes were G126, G114, G109 being G146 the most stable and early flowering accession. The highest number of branches (NOB) in SE-ME was recorded in genotypes G051, G174, G115, and G013 while G093 and G121 showed the lower number of branches (**Figure 7A**, B1). In NE-ME, G063, G114 and G126 were the genotypes with higher number of branches while G171 and the varieties Merkur, Merlin, Mistral and Fanfare revealed the smallest number of branches (**Figure 7A**, B2). For height of the first pod (HFP) and in both mega-environments the genotypes showed less stability. G100, G098 and G126 showed higher HFP in SE-ME (**Figure 7A**, C1) while G100, G298, G115 displayed the highest values in NE-ME (**Figure 7A**, C2).

The greatest number of pods per node (PPN) in the SE-ME was recorded in G171, G168 and G372 (**Figure 7B**, D1) while in NE-ME genotypes G029, Merkur and G171 were the most productive although unstable accessions (**Figure 7B**, D2). G116 and G076 yielded the lower number of PPN in SE-ME whereas G167, G126 and G165 were the least productive in NE-ME. The last trait with a repeatable pattern across years was seed number per pod (SPP). In SE-ME the genotype G025 was highly stable and productive followed by G048 and G023 that were less stable (**Figure 7B**, E1). On the other hand, G151, G091 and G029 were the most productive in NE-ME (**Figure 7B**, E2).

**Figure 8:**
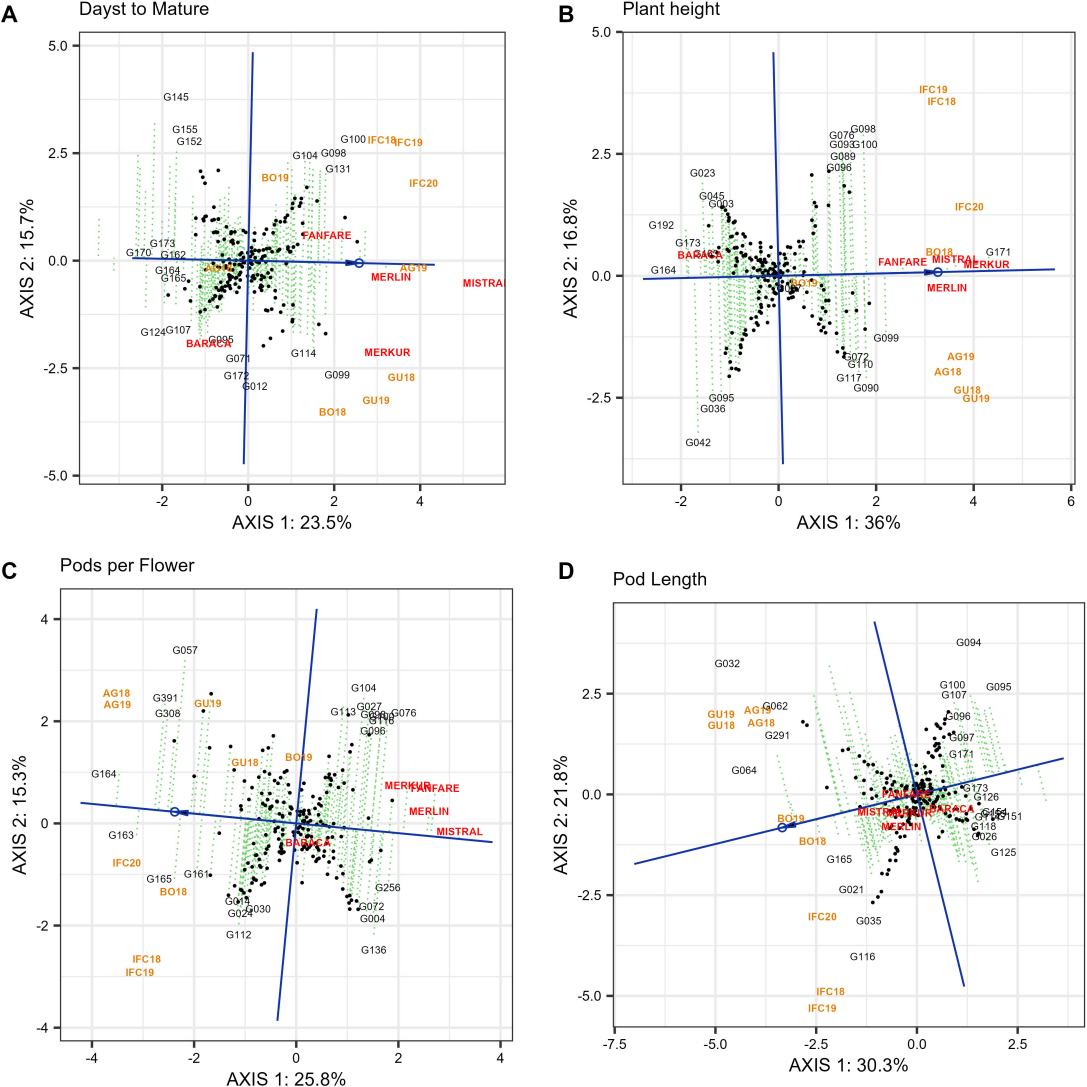
“Mean vs. stability” pattern of GGE biplot based of 220 Faba Bean genotypes in nine environments in four locations for days to mature **(A)**, plant height **(B)**, pods per flower **(C)** and pod length **(D)**. The biplots were based on centering = 0, SVP = 2, and scaling = 0.

For days to mature (DTM), plant height (PH), pods per flower (PPF) and pod length (PL), there was only one mega-environment since no patterns were repeatable across years. Unlike other late genotypes, cultivars Mistral, Merlin and Merkur take the longest period to mature (**Figure 8A**) being very stable. On the other hand, G170, G173, G162, G164 were highly stable and the first to mature (**Figure 8A**).

In case of plant height (PH), Merkur, Merlin, Mistral, Fanfare and G171 were highly stable and showed the tallest genotypes. The genotypes G164, along with G192, G173 and Baraca had the shortest plants (**Figure 8B**). The ranking of genotypes in case of pods per flower (PPF) (**Figure 8C**) were G164 > G163 > G391 > G308 > G057, showing the last three genotypes low stability. Otherwise, the cultivar Mistral (which was very stable), followed by Merlin, Fanfare, Merkur and G256, presented the smaller number of pods per flower. For pod length (PL), the best genotypes were G064 followed by G032, G291, G062 and G165 that showed the longest pods although low stability (**Figure 8D**).

The genotype ranking for the multi-trait stability index (MTSI) is displayed in **Supplementary Table S6**. Two traits were excluded from the analysis: day to full flowering and day to full maturity. These two traits have an excessively broad range of values, which covers up the other seven traits and leads to a low genotype ranking. Our ideotype was characterized by the following values: PH - 93.3 cm, NOB - 4.9, PL - 9.0 cm, HFP - 10 cm, PPN - 4.12, SPP - 8.33 and, PPF - 1. The genotypes that are most similar to the ideotype are highlighted in red in the **Supplementary Table S6**. Genotypes were chosen using a 15% selection intensity. Three analyses were conducted: the first ranked genotypes in the southern mega-environment, the second in the northern mega-environment applied on four traits (NOB, HFP, PPN, SPP) and the third included three traits (PH, PPF, PL) that had no effect on the separation of the mega-environments. Out of the 179 evaluated accessions in the SE-ME and NE-ME, 27 were selected with a 15% selection intensity for each environment. The most stable and best ranking genotypes in SE-ME are G018, G086, G081, G170 and G015 while in the north mega-environment are G091, G171, G177 (Merkur), G029 and G027. To select the best genotypes in a single mega-environment we used the information of three traits: PH, PPF and PL. As a result 33 out of 220 accessions were the ones closest to the ideotype. The best genotypes were G192, G039, G199, G175 and G075.

### 3.6 Cluster analysis

**Figure 9:**
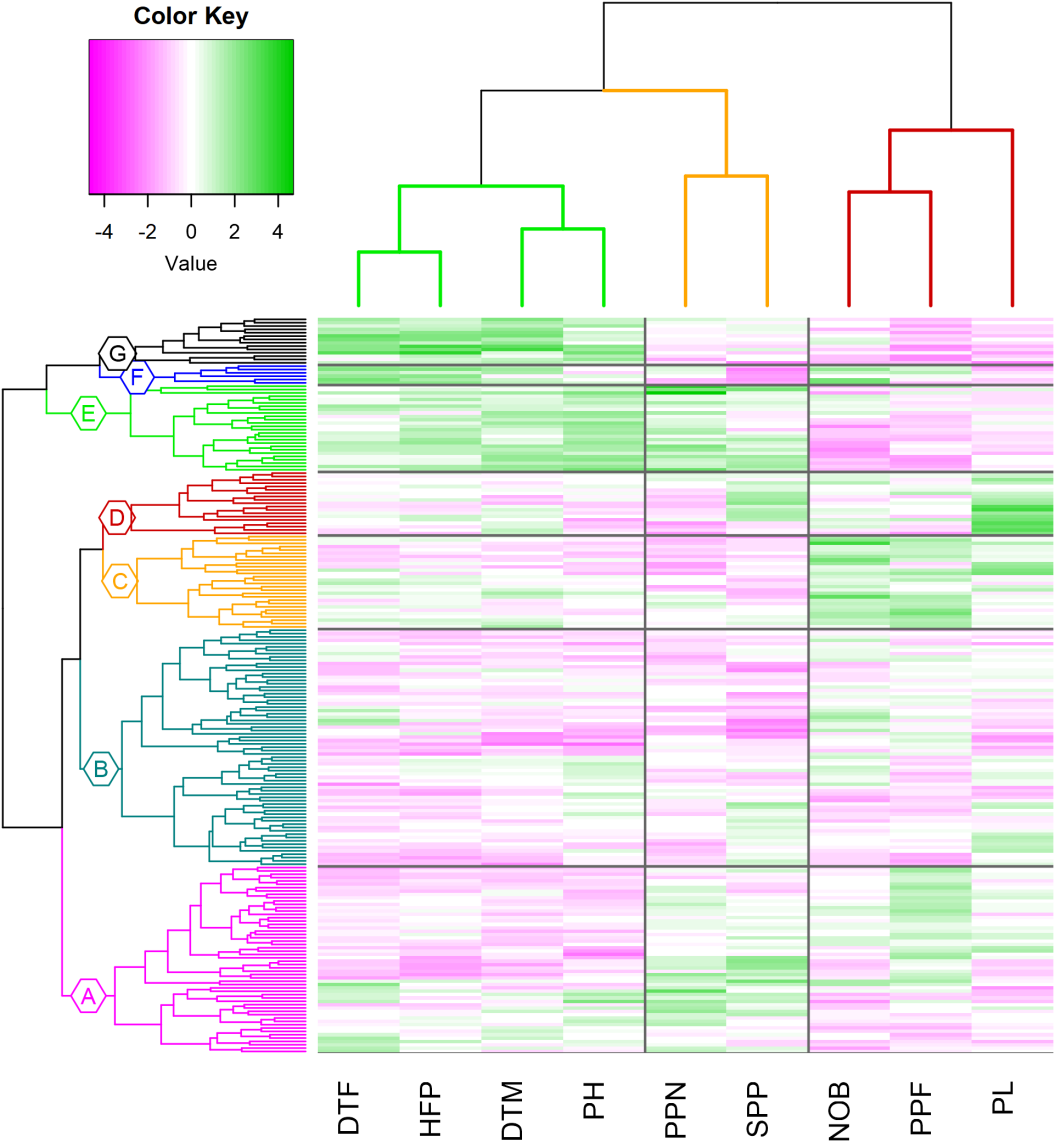
Dendrogram of the hierarchical clustering of 220 genotypes based on BLUP-s, using Euclidean distance and showing the phenotypic traits: DTF, days to flower; HFP, height of first pod; DTM, days to mature; PH, plant height; PPN, pods per node; SPP, seeds per pod; NOB, number of branches; PPF, Pods per flower; PL, pod length). The color scale is based on the value of the normalized mean for each trait: magenta (low) to green (high).

Hierarchical cluster analysis (HCA) was performed to group the genotypes sharing similar phenotypic traits (**Figure 9**). The 220 genotypes were grouped into two major clusters, where cluster (I) contained four subclusters: A, B, C and D, and the second major cluster (II), three subclusters: E, F and G. Subcluster B showed the maximum number of accessions (71), followed by subclusters A (56), C (28), E (26), D (19), G (14) and F (6) with the lowest number of accessions. The two major clusters are distinguished by four traits: days to flower (DTF), height of first pod (HFP), days to mature (DTM) and plant height (PH), such that cluster (I) is characterized by lower values for these four traits. Subclusters A and B gather accessions with the earliest flowering, and maturity date and smallest height of the first pod. On the other hand, subcluster C contains genotypes with the largest number of branches (NOB) and pods per flower (PPF), and subcluster D the longest pods (PL). The second major cluster, containing three sub-clusters (E, F and G), is characterized by high values for DTF, DTM, HFP and PH. Cluster F showed the largest values for DTF and HFP, while cluster G displayed the genotypes with later maturity. Subcluster E contains genotypes with highest plants (PH), largest number of pods per node (PPN) and largest number of seeds per pod (SPP). On the other hand, cluster G recorded the smallest number of PPF, cluster F had the smallest PL and SPP values and cluster E had the smallest number of branches (**Supplementary Table S7**).

The average diameter distance showed that the most diverse cluster was A (dist = 3.22) and the most compact was C (d = 2.92). The average linkage distance showed that the maximum inter-cluster distance was between clusters D and F (d = 5.87). The smallest inter-cluster distance in major cluster I was between A and B (dist = 3.45) while in major cluster II was between E and G (dist = 3.90) (**Supplementary Table S8**).

The complete collection was split into four groups based on the proportion of botanical types; however, part of the accessions was not characterized, so that this data is indicated as unknown (**Table 3**). Though all botanical types are presented in most subclusters, their relative abundance varies. Within subcluster A, which is distinguished by early flowering and smaller plant height, the equina type is the most prevalent (48%), as well as in subclusters B (48%) and D (50%), which share the same botanical type. The subclusters E and G are distinguished by the occurrence of minor types (65% and 72% respectively). In sub-cluster C, that includes the genotypes with the longest pods, the seeds are large and belong to the major and equina types. Equina is the most common seed type accounting for 41% of the genotypes. Minor accounts for 24%, followed by major types (17%) and only 5% of the collection were paucijuga types (**Table 3**).

### 3.7 Principal components analysis

**Figure 10:**
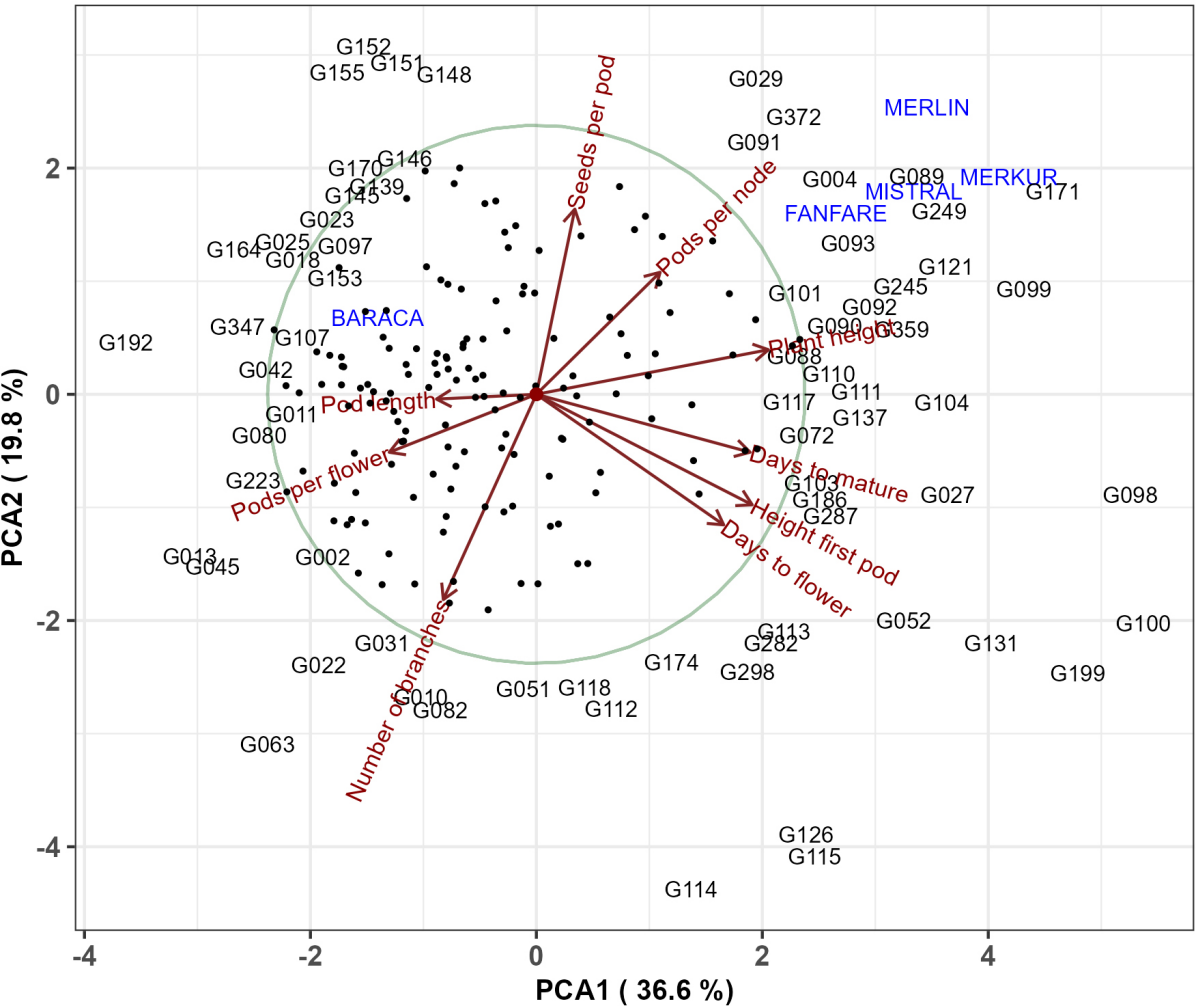
Principal component analysis of the 220 faba bean genotypes and nine phenotypic traits (days to flower, height of first pod, days to mature, plant height, pods per node, seeds per pod, number of branches, pods per flower and pod length). Genotypes were represented either with their names or with dots.

The principal component analysis revealed that the first two PCs explained 56.4% of the phenotypic variation among the 220 genotypes (**Figure 10**). The eigenvalues and proportion of the variation explained by the first three PCA loadings are shown in **Table 6**.

**Table 6:** The first three principal component scores for the nine agronomic traits.

	PC1	PC2	PC3
Eigenvalue	1.82	1.34	1.111
Proportion of variation	36.6	19.8	13.7
Component loadings			
DTF	0.38	-0.36	0.09
DTM	0.44	-0.16	-0.22
PH	0.47	0.12	-0.07
NOB	-0.19	-0.57	0.01
HFP	0.44	-0.31	-0.03
PPF	-0.30	-0.16	0.46
PPN	0.25	0.34	0.51
PL	-0.21	-0.01	-0.66
SPP	0.08	0.52	-0.20

DTF, days to flower; HFP, height of first pod; DTM, days to mature; PH, plant height; PPN, pods per node; SPP, seeds per pod; NOB, number of branches; PPF, Pods per flower; PL, pod length.

PC1 accounted for nearly 37% of the total variance observed. It was strongly related to plant height (PH), days to mature (DTM), height of first pod (HFP) and days to flowering (DTF) and was inversely related to pods per flower (PPF) and pod length (PL). PC2 (which accounted for 19.8% of the variance) was positively related to seeds per pod (SPP) and negatively related to the number of branches (NOB). PC3 explained 13.7% of the variation and was positively loaded by pods per flower (PPF) and pods per node (PPN) and negatively loaded by pod length (PL).

**Figure 11:**
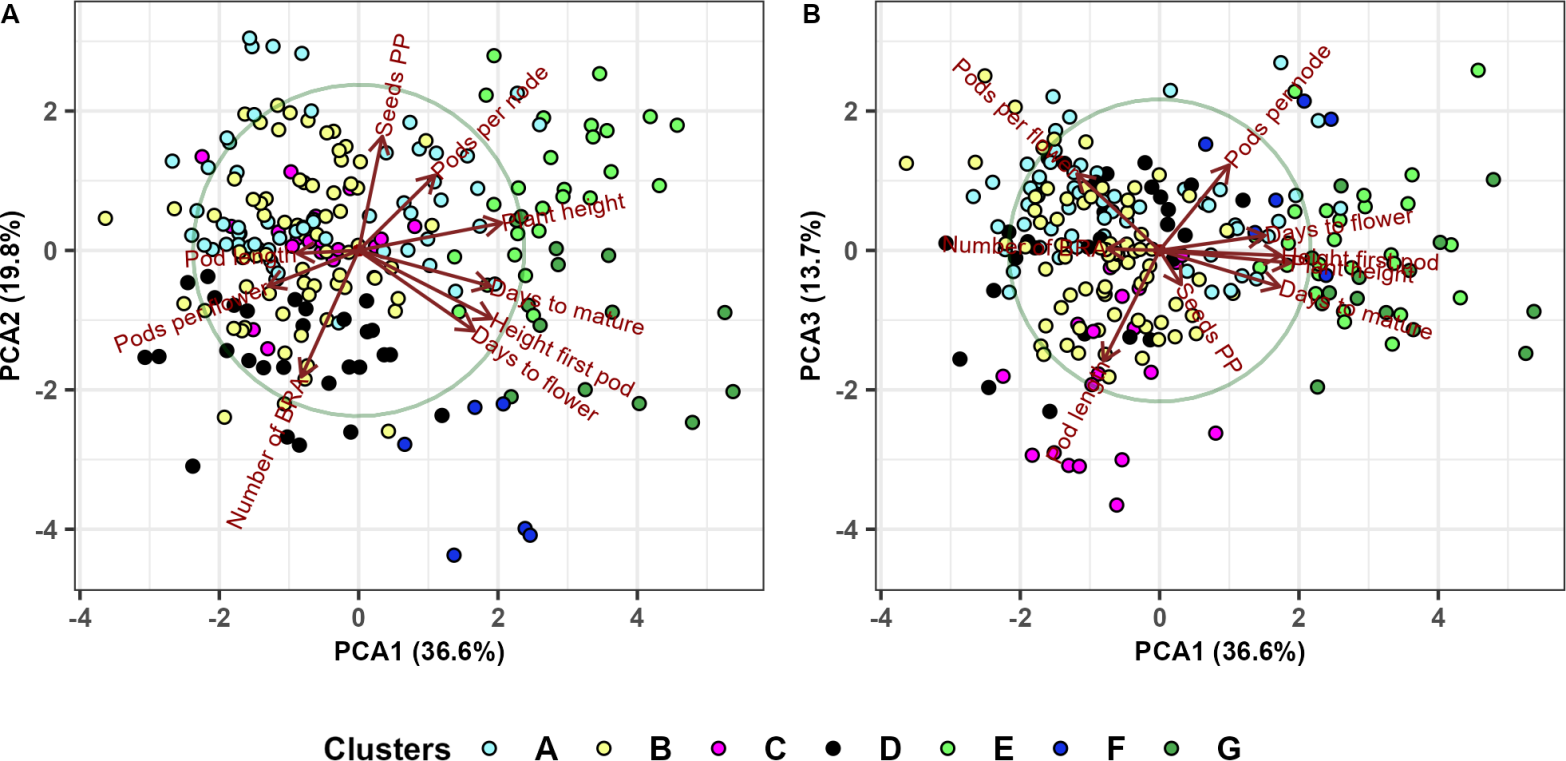
Principal component analysis of 220 faba bean genotypes and nine phenotypic traits (days to flower, height of first pod, days to mature, plant height, pods per node, seeds per pod, number of branches, pods per flower and pod length) based on the hierarchical clustering analysis. Subcluster names are as in section 3.6.

We used principal component analysis (**Figure 11**) to confirm and further explain the results obtained in the hierarchical clustering analysis (section 3.6). **Figure 11A** shows that the first PCA clearly separates the first two major clusters containing subclusters A, B, C and D from the second major cluster containing subclusters E, F and G. Subcluster G contains late genotypes with greater height of the first pod while subcluster E includes taller plants with larger number of pods per node. Subclusters A and B contain early and shorter genotypes, while subcluster C is characterized by a large number of branches. The third PCA axis (**Figure 11B**) separates cluster D (genotypes with large pod length), from clusters A, B and C.

**Figure 12:**
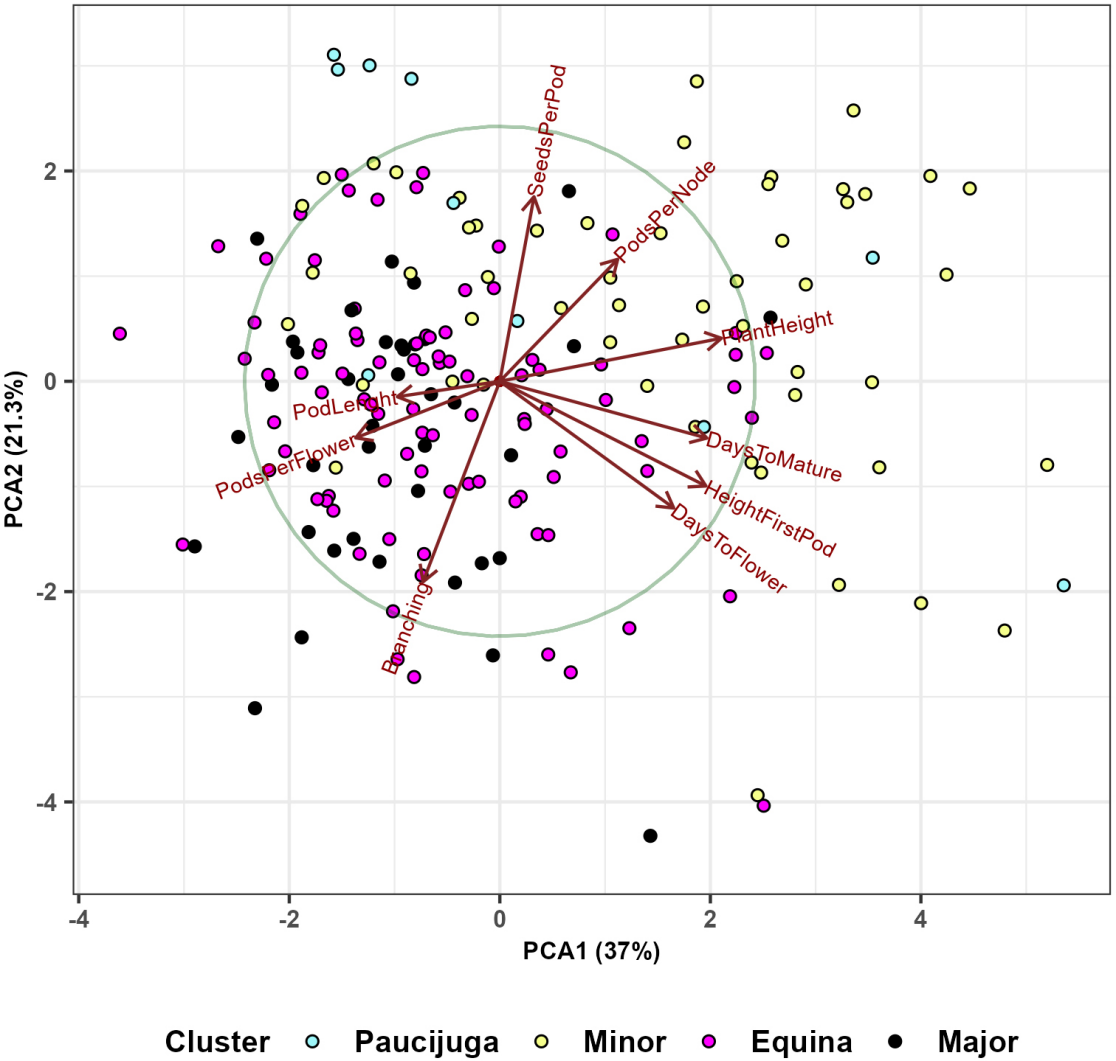
Principal component analysis based on 191 faba bean genotypes shows the relations between nine phenotypic traits (days to flower, height of first pod, days to mature, plant height, pods per node, seeds per pod, number of branches, pods per flower and pod length) and four botanical types (paucijuga, minor, equina, major). The analysis included genotypes with known botanical types.

We also used the principal component analysis to visualize the relationship between the nine phenotypic traits and the four botanical types (**Figure 12**); 37% of the variability is explained by the first principal component, and 21.3% by the second. The analysis distinguished the genotypes with longer flowering and maturation periods, larger plant heights, and higher first pods from the accessions with longer pod lengths and more pods per flower. The second component splits the collection primarily based on two characteristics: the number of seeds per pod and the number of branches.

## 4 Discussion

Faba bean is a low input protein crop that can adapt to a broad range of environmental conditions (Singh et al., 2013), however due to its high inter annual yield variability it is also recognized as an unstable crop (Link et al., 1995; Arbaoui et al., 2008; Cernay et al., 2015). In this study, a collection of 220 faba bean accessions was studied across four representative European regions and nine environments to evaluate the stability of yield components and to identify high yielding and stable genotypes suitable for each agroecological environment. The whole collection was evaluated for nine traits related to phenology, morphology, seed yield components, and botanical type using an augmented p-rep experimental design. To our knowledge, this is the largest faba bean collection analyzed so far, covering multiple locations and years to assess the magnitude of GEI and identify high yielding, and stable genotypes across a wide range of environments.

Faba bean is an open-field crop whose development, and seed yield is particularly susceptible to adverse weather conditions. As a drought-sensitive species (Parvin et al., 2019), in the Mediterranean region (Spain in our experiment) it is typically established in autumn (Link et al., 2010), while in the rest of European countries (Serbia, Belgium, Finland) it is sown in spring. Because of unpredictable climate changes (Milenković et al., 2023), the performances in the same location may vary year to year. The examples are environments IFC19 and IFC18 that had similar temperatures but IFC19 had lower levels of precipitation (**Supplementary Table S2B**) and as a consequence shorter ripening period. In Spain, where the ripening period is twice as long as in the other locations, the extended growing season resulted in a higher number of lateral branches (**Supplementary Table S4**). The combination of extended growing season and the high precipitation in AG19 (473.40 mm) (**Supplementary Table S2A**) resulted in the highest mean PH value (of 93 cm) with a maximum plant height of 154 cm, three times higher than the maximum value in BO19 (48 cm). Such noticeable development of the aerial parts, did not result in a positive effect on yield components. Thus, AG18 and AG19 together with BO19 were the environments with the lowest values in number of pods per flower and pods per node, while the number of seeds per pod had similar values ​​in all the localities. These outcomes are in agreement with Pilbeam et al. (1990), who reported that lateral branches are reproductively inferior compared to main stems; while other studies (Tuttobene et al, 1995; Barry and Storey, 1979) state that pods drop at high density due to competition for resources between vegetative and reproductive sinks (Pate and Armstrong, 1996). In the Nordic regions early maturation is a desired faba bean trait although very often is negatively correlated with seed yield (Skovbjerg et al., 2020). Similarly, significant negative correlations between DTF and DTM with seed yield components (PPF, PPN, SPP) were detected (**Figure 4**). Both phenological traits showed the lowest CV across all environments (DTF below 20% and DTM below 10%).

Developing environmentally resilient faba bean cultivars will depend on the genetic diversity available in the crop collections (Karkanis et al., 2018). The multi trial mixed model variances revealed that in our study the main variations were genotypic, particularly for morphological traits such as plant height (PH), height of the first pod (HFP), and pod length (PL). GEI affected most of the traits studied (**Table 4**), especially the period of maturation (DTM, 47.0%) and in traits representing pods setting (PPN, PPF and SPP), were the variance explained reached 46.6% 44.9% and 31.2%, respectively. This high GEI complicates significantly the faba bean breeding process. Hadou el hadj et al. (2022) revealed a high GEI effect on 15 productive, morphological, and phenological traits ranging from 74.08% to 83.21% while Skovbjerg et al. (2020) found that location and year effects accounted for 89% of the total seed yield variation while GEI was were small (2.4%). As mentioned above, in our collection, seed yield components were highly influenced by the environment. Broad-sense heritability (H2) is very often calculated by plant breeders to quantify the precision of MET (Covarrubias-Pazaran, 2021). In our multi-trail experiment, H2 for all nine traits was high, above 0.7 and for plant height and pod length above 0.9.

Plant height was affected not only by the different environments but also by the botanical types. In agreement with Link et al. (1995), minor types were the tallest genotypes, not only in Spain but in all environments.

Considering the seed size, it was noticeable that NOB and PL average values were significantly and positively different for minor, equina and major types in comparison with paucijuga lines. It was also observed that plants with larger seeds increased the number of branches and the length of its pods. In addition, PPN and SPP were significant and negatively affected by the seed size. (**Table 5**). This statement was also evident in the PCA analysis (**Figure 12**) which grouped larger seed genotypes according to pod length (PL), number of pods per flower (PPF) and number of branches (NB). Minor types were considerably later in maturity and showed higher HFP than paucijuga types. Similarly, equina and major types were slightly earlier in flowering than paucijuga and minor types.

Correlations showed that DTF and DTM followed by PH and HFP were the highest and positive correlated traits (**Figure 4**). Considering the indeterminate faba bean growth habit (Ostberg, 2021), this result indicates that a longer vegetative period leads to an increase in these morphological traits. Similar findings were also described by Sharifi and Aminpana (2014) and Kumar et al. (2017) while other scientists have not reported these relationships. On the other hand, the yield related traits number of pods per flower (PPF), number of pods per node (PPN) and number of seed per pod (SPP) were negatively correlated with DTF and DTM. This outcome is in agreement with Alghamdi (2007). Finally, the positive correlation found between PH, PPN, PL and SPP was consistent with different studies (Maalouf et al., 2015; Papastylianou et al., 2021).

The correlation analyses across the nine environments were consistent for all the traits revealing significant positive correlations for all of them, except for PPN between BO19 and IFC18. PH was the most highly positively correlated trait between environments (excluding BO19). In contrast, PPN and SPP were the traits showing less statistically significant correlations between environments meaning that there was a high variability among genotypes and considerable GxE effects (**Figure 5**).

The use of a wide range of environments allows breeders to distinguish mega-environments and identify materials with broad adaptability or adapted to specific areas. Several statistical methods have been developed for evaluating genotypes stability with consistent performance across environments. Among them, multivariate techniques such as GGE biplots are one of the most popular and powerful tools for extracting patterns of interactions in multi-environment trials (Yan and Tinker, 2005; Dardanellia et al., 2006; Gauch et al., 2008) and has been widely used in different faba bean analyses of adaptability and stability (Gurmu et al., 2012; Tadele et al., 2020; Papastylianou et al., 2021; Abou-Khater et al., 2022; Greveniotis et al., 2023).

In our study the “which-won-where” plots revealed which groups of locations consistently shared the best set of genotypes across years. DTF, NOB, HFP, PPN and SPP allowed a clear differentiation in two mega environments namely South European mega environment (SE-ME) and North European mega environment (NE-ME).

In the case of DTF, G131 was the later flowering genotype in the SE-ME all the years, especially in Serbia while G126 was the later in NE-ME (especially in Belgium). G114 located in the polygon vertex and belonging to a small sector between mega-environments, was the latest flowering line in both mega environments.

The best lines in SE-ME were small seeded types while the winning genotypes in NE-ME were larger (major or equina types). The “which-won-where” biplot for NOB was divided into six sectors and also two mega environments were clearly defined. G051 was the winning genotype in SE-ME, or more precisely in the three IFC environments and G063 won in NE-ME while G115 and G114 were the most stable in each of these mega-environments. The standard cultivars Merkur, Fanfare and Mistral, as well as line G171 were the winning genotypes with fewer branches. In the case of height of first pod (HFP) the winning genotype in SE-ME was the minor-paucijuga genotype G100 while in NE-ME the best genotype was G126. G098 was the winner in both mega-environments. The genotype G171 was the best for PPN in SE-ME. Similarly, G029 was the winner in the NE-ME. Finally, G048 had the best performance for SPP in SE-ME, while in the second mega-environment was G151. The which-won-where biplots for DTM and DTF clearly differed in patterns and were especially affected by the climate in each location. No mega-environments were discernible as some of the environments were mixed. Cultivars Mistral in environment AG19 and Merkur in GU18 were the accessions with longer periods for maturation. Based on the definition of mega-environments, the geographical locations falling into each mega-environment can be used as test locations having high discriminating ability and representativeness for a specific morphological, phenological or yield related trait. According to our results DTF, NOB, HFP, PPN and SPP, are the traits for which the best performing genotypes can be selected in both the SE-ME and NE-ME. The repeatable patterns identified have important practical implications in faba bean breeding. Using ME-specific cultivars will convert the repeatable GE into genotypic main effect within ME, thereby improving heritability and selection gain and maximizing regional and overall productivity (Yan et al., 2023).

One of the main targets in faba bean breeding is to achieve yield stability under different environmental conditions (Bodner et al., 2018). To do so, yield and stability related traits must be considered simultaneously in multi-environment trials. Using the “mean vs. stability” view, the genotypes were arranged along the “average environment-axis” based on their average performance in all environments, with the arrow pointing to the highest value (**Figures 7A, B**). The most favorable accessions for early flowering were G164 and G146 in SE-ME and NE-ME, respectively while for late flowering were G131, G115 and G199 or G126 and G114 in SE-ME and NE-ME, respectively. Concerning number of branches (NOB) the best ones were G051 (in SE-ME) and G114 (in NE-ME). In case of height of first pod (HFP) all genotypes revealed a high instability in both mega-environments being G100 and G126 the ones with the highest values and G171 and G099 the most stable, respectively. The highest and most stable value for number of seeds per pod (SPP) in SE-ME was detected in G025 followed by G048, G023, G165 all belonging to equina or major types, the last ones known to be well adapted to Mediterranean conditions (Link et al., 1995). In the north mega-environment NE-ME, G151 was the accession with the highest seed per pod (SPP) value followed by G091.

Regarding plant height (PH), number of pods per flower (PPF), pod length (PL) and days number to full maturity (DTM), only one mega-environment was detected, since none of the locations (environments) shared the best set of genotypes across years (Yan and Rajcan, 2002). The latest genotypes for DTM were the standard cultivars Mistral, Merlin, Merkur and Fanfare and, except Merkur, all were stable. The same cultivars together with G171 were the most favorable lines for PH in all environments. G164 was the shortest in the whole collection and together with G163, revealed the highest number of pods per flower (PPF) while the standard cultivars showed the lowest number of PPF.

Our results suggest that some genotypes (e.g. G114 and G115) had quite stable performance based on more than one trait (DTF and NOB) in a wide range of climatic conditions. Additionally, G114 was the best line with wider adaptation to both mega-environments and could be recommended for wider production in similar regions. Similarly, G100 and G126, although less stable, revealed high performance for DTF and HFP in the SE-ME and the NE-ME, respectively. Similar outcomes were reported in faba bean studies by Milioli et al. (2018) and Abou-Khater et al. (2022) or Greveniotis et al. (2023), indicating that more than one trait should be used to characterize the performance of genotypes across environments and enable more reliable selection and recommendation of genotypes.

The architecture modifications in the European faba bean breeding programs are, in general, directed toward a compact ideotype with synchrony in the reproductive development and reduced vegetative growth. Thus, taller plants with an excessive number of branches (high NOB and PH) are not desired since it results in lodging problems. Concerning HFP, the ideal plants have a continuous podding starting at 10-15 cm from the ground to avoid losses with the mechanical harvesting. Obviously, plants with the highest PPN, SPP and PPF values are the best as they will produce the highest yields. Finally, the ideal pod length (PL) will depend on the region and use of these pods (human consumption or animal feeding). We designed the ideal genotype based on these requirements and the data obtained in the different field evaluations. Thus, for PPN, SSP and PPF we considered the maximum values across all environments; for HFP 10 cm; for NOB, PH and PL the highest mean values across all the environments (**Supplementary Table S4**). The botanical type of the accessions is provided in **Supplementary Table S1**, where we can observe that the most stable genotypes in the southern environment correspond to the equina botanical type, whereas the minor seed type was the best in the northern mega-environment. When comparing the MTSI results for the SE-ME and NE-ME, it was evident that the genotypes tested in the south were more similar to the ideal genotype, revealing lower MTSI values.

In order to facilitate machine harvesting minimizing yield loss, a faba bean ideotype was developed in response to the demands of the European agricultural sector. This is a theoretical plant model which combines the most desirable characteristics and that, in principle, can attain maximum yield within a given environment. However, the agronomic practices together with abiotic and biotic factors may greatly affect those traits (Carbajal-Friedrich and Burgess, 2024) making it difficult to achieve. Our outcomes bring up the idea that the concept of ideotype should not be considered as static, since it needs to combine the available gene information controlling complex traits with the knowledge on the G x E interactions affecting crop performance. Nevertheless, the stability findings reported here may help to build the ideotype in future faba bean breeding programs. Moreover, strategies such as marker-assisted recurrent selection (MARS) based on QTL analyses (Gutiérrez et al., 2013; Kaur et al., 2014; Atienza et al., 2016; Catt et al., 2017; Ocaña-Moral et al., 2017; Sudheesh et al., 2019; Gutiérrez and Torres, 2021; Aguilar-Benítez et al., 2022; Aguilar-Benitez et al., 2021), and GWAS-genome-wide association studies (Gutiérrez et al., 2023, Gutiérrez and Torres, 2021; Maalouf et al., 2022; Ali et al., 2016; Skovbjerg et al., 2023; Jayakodi et al., 2023) are valuable tools available today in this crop. With this improved access to genetic markers, genomic selection presents a powerful strategy to improve trait selection in future faba bean breeding programmes.

The hierarchical cluster analysis (HCA) grouped the 220 faba bean lines into two mega-clusters with seven subclusters. The first mega-cluster (including subclusters A to D) was characterized by genotypes with lower values in the phenological traits DTF and DTM, plant height (PH) and height of first pod (HFP), most of these genotypes were major and equina types (**Table 3**). The PCA analysis (**Figure 10**) detected genotypes with high performances surpassing the checks. Thus, apart from Merkur, Merlin and Mistral, genotypes G171, G089, G249 and G099 achieved the best performances for PH and PPN. Considering the phenological traits (DTF and DTM) and HFP, genotypes G100, G199, G131 were the ones requiring more time for development, although the performance in yield-related traits was quite low. Conversely, G192, G164 and G025 were among the best genotypes for PL and SPP. The comparison of the PCA analyses in **Figures 10**, **12** allowed us to infer the botanical type to which these genotypes belong. Thus, in accordance with the cluster analysis, equina and major types are mainly characterized by larger values of PL, PPF and NOB, while minor types showed, in general, larger values for the remaining six traits.

As previously stated, the hierarchical cluster analysis (HCA) and the principal component analysis (PCA) grouped the genotypes based on the nine evaluated traits. HCA identified seven subclusters and the percentage of botanical types (paucijuga, minor, equina and major) in each subcluster and in the whole collection are shown in **Table 3**. Similarly, the PCA analyses, first separated the samples according to the evaluated traits and then the accessions were assigned to their botanical type (**Figure 12**). Results in both analyses lead to the same conclusions, when the starting breeding material is a minor type, with a high probability, we will obtain tall plants. On the other hand, major types will produce short plants with long pods and a large number of pods per flower (**Figure 12**). Considering that the analysis included a set of 220 genotypes and nine environments, we can argue that the selection of the botanical type is a key starting point in faba bean breeding programs.

Finally, we would like to underline the advantages and disadvantages of the experimental design used in this study. The main advantage of augmented designs over complete block designs (such as a completely randomized design and a randomized complete block design) is that it requires less experimental resources (seeds, manpower and management costs). If the researchers lack sufficient seeds, and are interested in comparing new entries to the controls, an augmented design would be a good choice. Thus, augmented design is especially useful when large numbers of genotypes are to be evaluated, and should be used for selection in the early stages of breeding programs (Aguade, 2011). The main disadvantage of augmented design over complete block designs is that it has less precision for comparing unreplicated treatments and relatively few degrees of freedom for experimental error, which reduces the power to detect differences among treatments. Some other disadvantages of using augmented design is that missing data cannot be calculated, data analysis is complex, and it can be used only in single-factor experiments (Sewenet, 2019).

## 5 Conclusions

The analysis of 220 faba bean genotypes in four European countries based on five traits (DTF, NOB, HFP, PPN and SPP) distinguished two mega-environments (North European ME and South European ME). Using the “which won where” biplot, winning genotypes were identified in each mega-environment and using the mean vs. stability plots, winning and several highest performing genotypes were distinguished. The analyses identified genotypes with better performances compared with commercial varieties and winning genotypes according to the highest trait value in each environment. In line with our results it is not possible to create cultivars suitable for both ME with equally good performances. Both ME are suitable as a region to perform faba bean test locations. The augmented p-rep design showed to be effective for multi-location trials with a large number of genotypes and the MTSI index emerged as a reliable method for identifying genotypes with high stability and performance across multiple traits. Hierarchical cluster analysis and principal component analyses showed a clear correlation between the traits analyzed and the botanical type. These findings indicate that botanical type is one of the most significant seed traits affecting development in each environment, and it must be taken into account in faba bean breeding activities. Collecting data from 220 genotypes across nine environments was a very time and labor-intensive effort where high-throughput phenotyping would be an advantageous alternative to validate findings, particularly for phenological features. The information derived from this study provides a chance for breeding new resilient faba bean cultivars adapted to different agroecological European regions, a critical point for addressing our reliance on protein imports, to enhance sustainable agricultural practices and to increase the production of protein-rich crops within Europe.

## Data Availability

The original contributions presented in the study are included in the article/**Supplementary Material**, further inquiries can be directed to the corresponding author/s.

## Footnotes

1
https://eur-lex.europa.eu/legalcontent/EN/TXT/HTML/?uri=CELEX%3A52018DC0757.

2
https://www.eea.europa.eu/data-and-maps/figures/biogeographical-regions-ineurope-1.

## References

[B1] Abebe T. Nega Y. Mehari M. Mesele A. Workineh A. Beyene H. (2015). Genotype by environment interaction of some faba bean genotypes under diverse broomrape environments of Tigray, Ethiopia. J. Plant Breed. Crop Sci. 7, 79–86. doi: 10.5897/JPBCS2014.0493

[B2] Abou-Khater L. Maalouf F. Jighly A. Rubiales D. Kumar S. (2022). Adaptability and Stability of Faba Bean (Vicia faba L.) Accessions under Diverse Environments and Herbicide Treatments. Plants 11, 251. doi: 10.3390/plants11030251 35161237 PMC8839948

[B3] Adekiya A. O. Agbede T. M. Aboyeji C. M. Dunsin O. Ugbe J. O. (2017). Green manures and NPK fertilizer effects on soil properties, growth, yield, mineral and vitamin C composition of okra (Abelmoschus esculentus (L.) Moench). J. Saudi Soc Agric. Sci. 18, 218–223. doi: 10.1016/j.jssas.2017.05.005

[B4] Aguade A. (2011). Construction and analysis of Augmented and modified augmented Design (Addis Ababa, Ethiopia: Addis Ababa University). Doctoral thesis.

[B5] Aguilar-Benitez D. Casimiro-Soriguer I. Maalouf F. Torres A. M. (2021). Linkage mapping and QTL analysis of flowering time in faba bean. Sci. Rep. 11, 13716. doi: 10.1038/s41598-021-92680-4 34215783 PMC8253854

[B6] Akinyosoye S. T. Agbeleye O. A. Adetumbi J. A. Ukachukwu P. C. Amusa O. D. (2023). Genotype–genotype × environment (GGE) biplot analysis of winged bean for grain yield. Acta Hortic. Regiotecturae 26, 53–63. doi: 10.2478/ahr-2023-0009

[B7] Alghamdi S. S. (2007). “Genetic behavior of some selected faba bean genotypes.” in Proceedings of African Crop Science Conference (El-Minia, Egypt: African Crop Science Society), 8, 709–714.

[B8] Alghamdi S. S. Migdadi H. M. Ammar M. H. Paull J. G. Siddique K. (2012). Faba bean genomics: current status and future prospects. Euphytica 186, 609–624. doi: 10.1007/s10681-012-0658-4

[B9] Ali M. B. M. Welna G. C. Sallam A. Martsch R. Balko C. Gebser B.. (2016). Association analyses to genetically improve drought and freezing tolerance of faba bean (Vicia faba L.). Crop Sci. 56, 1036–1048. doi: 10.2135/cropsci2015.08.0503

[B10] Araújo M. S. de Aragao W. F. L. dos Santos S. P. Freitas T. K. T. Saraiva V. C. Damasceno-Silva K. J.. (2022). Evaluation of adaptability and stability for iron, zinc and protein content in cowpea genotypes using GGE biplot approach. Heliyon 8, e11832. doi: 10.1016/j.heliyon.2022.e11832 36506391 PMC9732134

[B11] Arbaoui M. Balko C. Link W. (2008). Study of faba bean (Vicia faba L.) winter-hardiness and development of screening methods. Field Crop Res. 106, 60–67. doi: 10.1016/j.fcr.2007.10.015

[B12] Atienza S. G. Palomino C. Gutiérrez N. Alfaro C. M. Rubiales D. Torres A. M.. (2016). QTLs for ascochyta blight resistance in faba bean (Vicia faba L.): validation in field and controlled conditions. Crop Pasture Sci. 67, 216–224. doi: 10.1071/CP15227

[B13] Barry P. Storey T. S. (1979). Influence of some cultural practices on the yield, development and quality of field beans (Vicia faba L.). Iran. J. Agr. Res. 18, 77–88.

[B14] Bates D. Mächler M. Bolker B. Walker S. (2015). Fitting linear mixed-effects models using lme4. J. Stat. Software 67, 1–48. doi: 10.18637/jss.v067.i01

[B15] Bodner G. Kronberga A. Lepse L. Olle M. Vågen I. M. Rabante L.. (2018). Trait identification of faba bean ideotypes for Northern European environments. Eur. J. Agron. 96, 1–12. doi: 10.1016/j.eja.2018.02.008

[B16] Carbajal-Friedrich A. A. J. Burgess A. J. (2024). The role of the ideotype in future agricultural production. Front. Plant Physiol. 2. doi: 10.3389/fphgy.2024.1341617

[B17] Catt S. C. Braich S. Kaur S. Paull J. G. (2017). QTL detection for flowering time in faba bean and the responses to ambient temperature and photoperiod. Euphytica 213, 125. doi: 10.1007/s10681-017-1910-8

[B18] Cernay C. Ben-Ari T. Pelzer E. Meynard J. M. Makowski D. (2015). Estimating variability in grain legume yields across Europe and the Americas. Sci. Rep. 5, 11171. doi: 10.1038/srep11171 26054055 PMC4459183

[B19] Covarrubias-Pazaran E. G. (2021). Heritability: meaning and computation. 1st ed. Montpellier, France: CGIAR Excellence in Breeding Platform (EiB).

[B20] Crépon K. Marget P. Peyronnet C. Carrouée B. Arese P. Duc G. (2010). Nutritional value of faba bean (Vicia faba L.) seeds for feed and food. Field Crops Res. 115, 329–339. doi: 10.1016/j.fcr.2009.09.016

[B21] Cullis B. R. Smith A. B. Coombes N. E. (2006). On the design of early generation variety trials with correlated data. J. Agricultural Biological Environ. Stat 11, 381. doi: 10.1198/108571106X154443

[B22] Dardanellia J. L. Balzarinic M. Martíneza M. J. Cunibertib M. Resnikd S. Ramundaa S. F.. (2006). Soybean maturity groups, environments, and their interaction define mega-environments for seed composition in Argentina. Crop Sci. 46, 1939 –11947. doi: 10.2135/cropsci2005.12-0480

[B23] De Visser C. Schreuder R. Stoddard F. (2014). The EU’s dependence on soya bean import for the animal feed industry and potential for EU produced alternatives. Oilseeds fats Crops Lipids 21, D407. doi: 10.1051/ocl/2014021

[B24] Duc G. Aleksić J. M. Marget P. Mikic A. Paull J. Redden R. J.. (2015). Faba bean. Grain legumes. New York: Springer, 141–178. doi: 10.1007/978-1-4939-2797-5_5

[B25] Elias A. A. Robbins K. R. Doerge R. W. Tuinstra M. R. (2016). Half a century of studying genotype × Environment interactions in plant breeding experiments. Crop Sci. 56, 2090. doi: 10.2135/cropsci2015.01.0061

[B26] FAOSTAT (2021). World food and agriculture - statistical yearbook 2021 (Rome: Food and Agriculture Organization of the United Nations). Available at: https://www.fao.org/3/cb4477en/cb4477en.pdf. doi: 10.4060/cb4477en

[B27] FAOSTAT (2022). World food and agriculture – statistical yearbook 2022. Available online at: https://www.fao.org/3/cc2211en/cc2211en.pdf.

[B28] Fernandez-Aparicio M. Sillero J. C. Rubiales D. (2007). Intercropping with cereals reduces infection by Orobanche crenata in legumes. Crop Prot. 26, 1166–1172. doi: 10.1016/j.cropro.2006.10.012

[B29] Flores F. Hybl M. Knudsen J. C. Marget P. Muel F. Nadal S.. (2013). Adaptation of spring faba bean types across European climates. Field Crops Res. 145, 1–9. doi: 10.1016/j.fcr.2013.01.022

[B30] Gabriel K. R. (1971). The biplot graphic display of matrices with application to principal component analysis. Biometrika 58, 453–467. doi: 10.1093/biomet/58.3.453

[B31] Galili T. (2015). Dendextend: an R package for visualizing, adjusting and comparing trees of hierarchical clustering. Bioinformatics 31, 3718–3720. doi: 10.1093/bioinformatics/btv428 26209431 PMC4817050

[B32] Gauch H. G. (2006). Statistical analysis of yield trials by AMMI and GGE. Crop Sci. 46, 1488–1500. doi: 10.2135/cropsci2005.07-0193

[B33] Gauch H. G. Piepho H. P. Annicchiarico P. (2008). Statistical analysis of yield trials by AMMI and GGE: further considerations. Crop Sci. 48, 866–889. doi: 10.2135/cropsci2007.09.0513

[B34] Gauch H. G. Zobel R. W. (1996). “AMMI analysis of yield trials,” in Genotype-by-environment interaction. Eds. Kang M. S. Gauch H. G. (CRC Press, Boca Raton), 85–122. doi: 10.1201/9781420049374.ch4

[B35] Gela T. S. Khazaei H. Podder R. Vandenberg A. (2023). Dissection of genotype-by-environment interaction and simultaneous selection for grain yield and stability in faba bean (Vicia faba L.). Agron. J. 115, 474–488. doi: 10.1002/agj2.21268

[B36] Gnanasambandam A. Paull J. Torres A. Kaur S. Leonforte T. Li H.. (2012). Impact of molecular technologies on faba bean (Vicia faba L.) breeding strategies. Agronomy 2, 132–166. doi: 10.3390/agronomy2030132

[B37] Greveniotis V. Bouloumpasi E. Zotis S. Korkovelos A. Kantas D. Ipsilandis C. G. (2023). Genotype-by-environment interaction analysis for quantity and quality traits in faba beans using AMMI, GGE models, and stability indices. Plants 12, 3769. doi: 10.3390/plants12213769 37960125 PMC10648669

[B38] Gurmu F. Lire E. Asfaw A. Alemayehu F. Rezene Y. Ambachew D. (2012). GGE-biplot analysis of grain yield of faba bean genotypes in southern Ethiopia. Electronic J. Plant Breed. 3, 898–907.

[B39] Gutiérrez N. Palomino C. Satovic Z. Ruiz-Rodríguez M. D. Vitale S. Gutiérrez M. V. (2013). QTLs for Orobanche spp. resistance in faba bean: identification and validation across different environments. Mol. Breed. 32, 909–922. doi: 10.1007/s11032-013-9920-2

[B40] Gutiérrez N. Torres A. M. (2021). QTL dissection and mining of candidate genes for Ascochyta fabae and Orobanche crenata resistance in faba bean (Vicia faba L.). BMC Plant Biol. 21, 551. doi: 10.1186/s12870-021-03335-5 34809555 PMC8607628

[B41] Hadou el hadj D. Tellah S. Goumeida K. Aitouakli S. Tifest C. Ammi N.. (2022). Evaluation of adaptability of different faba bean landraces under mediterranean field conditions of central-northern Algeria. Agronomy 12, 1660. doi: 10.3390/agronomy12071660

[B42] Haisirikul P. Sontornkarun T. Burakorn W. Pinta W. Chankaew S. Monkham T.. (2020). Yield performance of early-maturity cowpea (Vigna unguiculata) elite lines under four varied environments. Thai J. Agric. Sci. 53, 165–177.

[B43] Harrell C. O. (2023). “Hmisc: harrell miscellaneous,” in R package version 5.0.1, CRAN.R-project.org/package=Hmisc.

[B44] Hassani M. Mahmoudi S. B. Saremirad A. Telaghani D. (2023). Genotype by environment and genotype by yield*trait interactions in sugar beet: analyzing yield stability and determining key traits association. Sci. Rep. 13, 23111. doi: 10.1038/s41598-023-51061-9 PMC10764822 38172529

[B45] Hauggaard-Nielsen H. Jørnsgaard B. Kinane J. Jensen E. (2008). Grain legume–cereal intercropping: The practical application of diversity, competition and facilitation in arable and organic cropping systems. Renewable Agric. Food Syst. 23, 3–12. doi: 10.1017/S1742170507002025

[B46] Henchion M. Hayes M. Mullen A. M. Fenelon M. Tiwari B. (2017). Future protein supply and demand: strategies and factors influencing a sustainable equilibrium. Foods 6, 53. doi: 10.3390/foods6070053 28726744 PMC5532560

[B47] Hongyu K. Penña M. G. Araújo L. B. Dias C. T. S. (2014). Statistical analysis of yield trials by AMMI analysis of genotype × environment interaction. Biometrical Lett. 51, 89–102. doi: 10.2478/bile-2014-0007

[B48] Jayakodi M. Golicz A. A. Kreplak J. Fechete L. I. Angra D. Bednář P.. (2023). The giant diploid faba genome unlocks variation in a global protein crop. Nature 615, 652–659. doi: 10.1038/s41586-023-05791-5 36890232 PMC10033403

[B49] Johnson R. Johnston W. Nelson M. Simon C. Golob C. (1999). “Core utilization and development–an example with Poa pratensis,” in Core collections for today and tomorrow, vol. 49–60 . Eds. Johnson R. C. Hodgkin T. (Plant Genetic Resources Institute, New York, NY).

[B50] Karkanis A. Ntatsi G. Lepse L. Fernández J. A. Vågen I. M. Rewald B.. (2018). Faba bean cultivation–revealing novel managing practices for more sustainable and competitive European cropping systems. Front. Plant Sci. 1115. doi: 10.3389/fpls.2018.01115 PMC6083270 30116251

[B51] Kassambara A. (2022). ggcorrplot: Visualization of a Correlation Matrix using ‘ggplot2’. R package version 0.1.4. Available online at: https://CRAN.R-project.org/package=ggcorrplot.

[B52] Kaur S. Kimber R. B. E. Cogan N. O. I. Materne M. Forster J. W. Paull J. G. (2014). SNP discovery and high-density genetic mapping in faba bean (Vicia faba L.) permits identification of QTLs for ascochyta blight resistance. Plant Sci. 217–218, 47–55. doi: 10.1016/j.plantsci.2013.11.014 24467895

[B53] Koç S. Orak A. Tenikecier H. S. Saglam N. (2018). Relationship between seed yield and yield characteristics in faba bean (Vicia faba L.) by GGE-biplot analysis. J. Life Sci. 12, 105–110. doi: 10.17265/1934-7391/2018.02.005

[B54] Kumar P. Das R. R. Bishnoi S. K. Vinay S. (2017). Inter-correlation and path analysis in faba bean (Vicia faba L.). Electronic J. Plant Breed. 8, 395–397. doi: 10.5958/0975-928X.2017.00059.X

[B55] Kuznetsova A. Brockhoff P. B. Christensen R. H. B. (2017). lmerTest package: tests in linear mixed effects models. J. Stat. Software 82, 1–26. doi: 10.18637/jss.v082.i13

[B56] Lahti L. Huovari J. Kainu M. Biecek P. (2017). Retrieval and analysis of Eurostat open data with the eurostat package. R J. 9, 385–392. doi: 10.32614/RJ-2017-019 Package URL: http://ropengov.github.io/eurostat Article URL: https://journal.r-project.org/archive/2017/RJ-2017-019/index.html

[B57] Lal R. K. Chanotiya C. S. Mishra A. (2022). Mega-environment investigation based on the GGE biplot and genotype selection for high essential oil yield in vetiver grass (Chrysopogon zizanioides L. Roberty) Acta Ecologica Sin. 42, 542–552. doi: 10.1016/j.chnaes.2022.01.002

[B58] Link W. Balko C. Stoddard F. L. (2010). Winter hardiness in faba bean: physiology and breeding. Field Crop Res. 115, 297–307. doi: 10.1016/j.fcr.2008.08.004

[B59] Link W. Dixkens C. Singh M. Schwall M. Melchinger A. E. (1995). Genetic diversity in European and Mediterranean faba bean germplasm revealed by RAPD markers. Theoret. Appl. Genet. 90, 27–32. doi: 10.1007/BF00220992 24173780

[B60] Liu X. Q. Rong J. Y. Liu X. Y. (2008). Best linear unbiased prediction for linear combinations in general mixed linear models. J. Multivariate Anal. 99, 1503–1517. doi: 10.1016/j.jmva.2008.01.004

[B61] Lyu J. I. Ramekar R. Kim J. M. Hung N. N. Seo J. S. Kim J. B.. (2021). Unraveling the complexity of faba bean (Vicia faba L.) transcriptome to reveal cold-stress-responsive genes using long-read isoform sequencing technology. Sci. Rep. 11, 21094. doi: 10.1038/s41598-021-00506-0 34702863 PMC8548339

[B62] Maalouf F. Abou-Khater L. Babiker Z. Jighly A. Alsamman A. M. Hu J.. (2022). Genetic dissection of heat stress tolerance in faba bean (Vicia faba L.) using GWAS. Plants 11 (9), 1108. doi: 10.3390/plants11091108 35567109 PMC9103424

[B63] Maalouf F. Nachit M. Ghanem M. E. Singh M. (2015). Evaluation of faba bean breeding lines for spectral indices, yield traits and yield stability under diverse environments. Crop Pasture Sci. 66, 1012–1023. doi: 10.1071/CP14226

[B64] Maalouf F. S. Suso M. J. Moreno M. T. (2002). Comparative performance of faba bean synthetics developed from different parental numbers. J. Genet. Breed. 56, 251–258.

[B65] Massicotte P. South A. (2023). rnaturalearth: World Map Data from Natural Earth. R package version 0.3.2. Available online at: https://CRAN.R-project.org/package=rnaturalearth.

[B66] Memon J. Patel R. Parmar J. D. Kumar S. Patel A. N. Patel N. B.. (2023). Deployment of AMMI, GGE-biplot and MTSI to select elite genotypes of castor (Ricinus communis L.). Heliyon 9, (2). doi: 10.1016/j.heliyon.2023.e13515 PMC9975251 36873144

[B67] Milenković J. Petrović M. Andjelković S. Mitra D. Singhal R. K. (2023). “Forage cultivation under challenging environment,” in Molecular Interventions for Developing Climate-Smart Crops: A Forage Perspective. Eds. Ahmed S. Pandey S. Chand S. (Springer, Singapore). doi: 10.1007/978-981-99-1858-4_8

[B68] Milioli A. S. Zdziarski A. D. Woyann L. G. Santos R. D. Rosa A. C. Madureira A.. (2018). Yield stability and relationships among stability parameters in soybean genotypes across years. Chil. J. Agric. Res. 78, 299–309. doi: 10.4067/S0718-58392018000200299

[B69] Moehring J. Williams E. R. Piepho. H.-P. (2014). Efficiency of augmented p-rep designs in multi-environmental trials. Theor. Appl. Genet. 127, 1049–1060. doi: 10.1007/s00122-014-2278-y 24553963

[B70] Nardino M. Baretta D. Carvalho I. R. Olivoto T. Follmann D. N. Szareski V. J.. (2016). Restricted maximum likelihood/best linear unbiased prediction (REML/BLUP) for analyzing the agronomic performance of corn. Afr. J. Agric. Res. 11, 4864–4872. doi: 10.5897/AJAR2016.11691

[B71] Neugschwandtner R. Ziegler K. Kriegner S. Wagentristl H. Kaul H. P. (2015). Nitrogen yield and nitrogen fixation of winter faba beans. Acta Agriculturae Scandinavica Section B - Soil Plant Sci. 65, 658–666. doi: 10.1080/09064710.2015.1042028

[B72] Nieweglowski L. (2020). clv: Cluster Validation Techniques. R package version 0.3.2.2. Available online at: https://CRAN.R-project.org/package=clv.

[B73] Ocaña-Moral S. Gutiérrez N. Torres A. M. Madrid E. (2017). Saturation mapping of regions determining resistance to Ascochyta blight and broomrape in faba bean using transcriptome-based SNP genotyping. Theor Appl Genet. 130, 2271–2282. doi: 10.1007/s00122-017-2958-5 28791437

[B74] O’Sullivan D. M. Angra D. (2016). Advances in faba bean genetics and genomics. Front. Genet. 7. doi: 10.3389/fgene.2016.00150 PMC4993074 27597858

[B75] Olivoto T. Alessandro D. C. da Silva A. G. J. Sari G. B. Maria I. Diel I. M. (2019). Mean performance and stability in multi-environment trials I: combining features of AMMI and BLUP techniques. Agron. J. 111, 1–12. doi: 10.2134/agronj2019.03.0220

[B76] Olivoto T. Lúcio A. D. (2020). Metan: An R package for multi-environment trial analysis. Methods Ecol. Evol. 11, 783–789. doi: 10.1111/2041-210X.13384

[B77] Ostberg J. (2021). Vicia faba determinate and indeterminate inflorescence genotypes: comparison of genetic variation at TFL1 locus. Second cycle, A2E (Alnarp: SLU, Dept. of Plant Breeding). Available at: https://stud.epsilon.slu.se/16453/1/Ostberg_J_210215.pdf (Accessed December 5, 2023).

[B78] Papastylianou P. Vlachostergios D. N. Dordas C. Tigka E. Papakaloudis P. Kargiotidou A.. (2021). Genotype X Environment Interaction Analysis of Faba Bean (Vicia faba L.) for Biomass and Seed Yield across Different Environments. Sustainability 13, 2586. doi: 10.3390/su13052586

[B79] Parvin S. Uddin S. Tausz-Posch S. Fitzgerald G. Armstrong R. Tausz M. (2019). Elevated CO2 improves yield and N2 fixation but not grain N concentration of faba bean (Vicia faba L.) subjected to terminal drought. Environ. Exp. Bot. 165, 161–173. doi: 10.1016/j.envexpbot.2019.06.003

[B80] Pate J. S. Armstrong E. L. (1996). Pea in ‘Photo assimilate distribution in plants and crops’. Eds. Zamski E. Schaffer A. (New York: Marcel Dekker Inc.), 625–642.

[B81] Pietrzak W. Kawa-Rygielska J. Król B. Lennartsson P. R. Taherzadeh M. J. (2016). Ethanol, feed components and fungal biomass production from field bean (Vicia faba var. equina) seeds in an integrated process. Bioresource Technol. 216, 69–76. doi: 10.1016/j.biortech.2016.05.055 27233099

[B82] Pilbeam C. J. Duc G. Hebblethwaite P. D. (1990). Effects of plant population density on springsown field beans (Vicia faba L.) with different growth habits. J. Agric. Sci. 114, 19–33. doi: 10.1017/S0021859600070957

[B83] Progeno. (2020). Available online at: https://www.progeno.net.

[B84] R Core Team (2022). “R: A language and environment for statistical computing,” in R foundation for statistical computing. (Vienna, Austria). Available at: https://www.R-project.org/.

[B85] Rubiales D. Flores F. Emeran A. A. Kharrat M. Amri M. Rojas-Molina M. M.. (2014). Identification and multi-environment validation of resistance against broomrapes (Orobanche crenata and Orobanche foetida) in faba bean (Vicia faba). Field Crops Res. 166, 58–65. doi: 10.1016/j.fcr.2014.06.010

[B86] Salahin N. Alam M. K. Islam M. M. Naher L. Majid N. M. (2013). Effects of green manure and tillage practice on maize and rice yields and soil properties. Aust. J. Crop Sci. 7, 1901–1911.

[B87] Sewenet K. H. (2019). A Review on comparison of complete and incomplete block designs. J. Biology Agric. Healthcare 9 (9). doi: 10.7176/JBAH

[B88] Sharifi P. Aminpana H. (2014). A study on the genetic variation in some of faba bean genotypes using multivariate statistical techniques. Trop. Agric. 91, 87–97.

[B89] Singh A. K. Bharati R. C. Manibhushan N. C. Pedpati A. (2013). An assessment of faba bean (Vicia faba L.) current status and future prospect. Afr. J. Agric. Res. 8, 6634–6641. doi: 10.5897/AJAR2013.7335

[B90] Skovbjerg C. K. Angra D. Robertson-Shersby-Harvie T. Kreplak J. Keeble-Gagnère G. Kaur S.. (2023). Genetic analysis of global faba bean diversity, agronomic traits and selection signatures. Theor. Appl. Genet. 136, 114. doi: 10.1007/s00122-023-04360-8 37074596 PMC10115707

[B91] Skovbjerg C. K. Knudsen J. N. Füchtbauer W. Stougaard J. Stoddard F. L. Janss L.. (2020). Evaluation of yield, yield stability, and yield–protein relationship in 17 commercial faba bean cultivars. Legume Sci. 2, e39. doi: 10.1002/leg3.39

[B92] Smith A. Cullis B. R. Thompson R. (2005). The analysis of crop cultivar breeding and evaluation trials: An overview of current mixed model approaches. J. Agric. Sci. 143, 449–462. doi: 10.1017/S0021859605005587

[B93] Souza A. G. Daher R. F. Santana J. G. S. Ambrosio M. Nascimento N. R. Vidal A. K. F.. (2023). Adaptability and stability of black bean genotypes for Rio de Janeiro, by GGE biplot analysis. Crop Breed. Appl. Biotechnol. 23, e43972323. doi: 10.1590/1984-70332023v23n2a15

[B94] Stoddard F. L. (2017). Grain legumes: an overview. Eds. Donal M.-B. Stoddard F. L. Watson C. A.. (CABI Digital Library), 70–87. doi: 10.1079/9781780644981.0070

[B95] Sudheesh S. Kimber R. B. E. Braich S. Forster J. W. Paull J. G. Kaur S. (2019). Construction of an integrated genetic linkage map and detection of quantitative trait loci for ascochyta blight resistance in faba bean (Vicia faba L.). Euphytica 215, 42. doi: 10.1007/s10681-019-2365-x

[B96] Suso M. J. Moreno M. T. Melchinger A. E. (1999). Variation in out-crossing rate and genetic structure on six cultivars of Vicia faba L. as affected by geographic location and year. Plant Breed. 118, 347. doi: 10.1046/j.1439-0523.1999.00389.x

[B97] Tadele M. Mohammed W. Jarso M. (2020). Yield stability and genotype × Environment interaction of faba bean (Vicia faba L.). Int. J. Plant Breed. Crop Sci. 7, 833–846.

[B98] Taleghani D. Rajabi A. Saremirad A. Fasahat P. (2023). Stability analysis and selection of sugar beet (Beta vulgaris L.) genotypes using AMMI, BLUP, GGE biplot and MTSI. Sci. Rep. 13, 10019. doi: 10.1038/s41598-023-37217-7 37340073 PMC10281985

[B99] Temesgen T. Keneni G. Sefera T. Jarso M. (2015). Yield stability and relationships among stability parameters in faba bean (Vicia faba L.) genotypes. Crop J. 3, 258–268. doi: 10.1016/j.cj.2015.03.004

[B100] Tuttobene R. Vagliasindi C. AEP (1995). “Effects of plant density on flowering characteristics, growth and yield in “Sikelia” a new faba bean genotipe (Vicia faba L.) recently released,” in Improving production and utilisation of grain legumes : 2nd European Conference on Grain Legumes, Copenhagen, Denmark, 9–13 July, Vol. 172. (Paris: AEP).

[B101] Warnes G. Bolker B. Bonebakker L. Gentleman R. Huber W. Liaw A.. (2022). gplots: Various R Programming Tools for Plotting Data. R package version 3.1.3. Available online at: https://CRAN.R-project.org/package=gplots.

[B102] Yan W. (2001). GGE Biplot-A Windows application for graphical analysis of multi-environment trial data and other types of two-way data. Agron. J. 93, 1111–1118. doi: 10.2134/agronj2001.9351111x

[B103] Yan W. Hunt L. A. (2002). Biplot analysis of diallel data. Crop Sci. 42, 21–30. doi: 10.2135/cropsci2002.0021 11756249

[B104] Yan W. Hunt L. A. Sheng Q. Szlavnics Z. (2000). Cultivar evaluation and mega-environment investigation based on GGE biplot. Crop Sci. 40, 596–605. doi: 10.2135/cropsci2000.403597x

[B105] Yan W. Kang M. S. (2003). GGE Biplot Analysis: A graphical tool for breeders, geneticists, and agronomists (Boca Raton, FL: CRC Press).

[B106] Yan W. Kang M. S. Ma B. Woods S. Cornelius P. L. (2007). GGE biplot vs. AMMI analysis of genotype-by-environment data. Crop Sci. 47, 643–655. doi: 10.2135/cropsci2006.06.0374

[B107] Yan W. Nilsen K. T. Beattie A. (2023). Mega-environment analysis and breeding for specific adaptation. Crop Sci. 63, 480–494. doi: 10.1002/csc2.20895

[B108] Yan W. Rajcan I. (2002). Biplot evaluation of test sites and trait relations of soybean in Ontario. Crop Sci. 42, 11–20. doi: 10.2135/cropsci2002.1100 11756248

[B109] Yan W. Tinker N. A. (2005). An integrated biplot analysis system for displaying, interpreting, and exploring genotype × Environment interaction. Crop Sci. 45, 1004–1016. doi: 10.2135/cropsci2004.0076

[B110] Zanella R. Meira D. Zdziarski A. D. Brusamarello A. P. Oliveira P. H. Benin G. (2019). Performance of common bean genotypes as a function of growing seasons and technological input levels. Pesqui. Agropecu. Trop. 49, e54989. doi: 10.1590/1983-40632019v4954989

