# Supplementary material for: Genotype by environment interactions and phenotypic traits stability of the EUCLEG faba bean collection
Source: Front Plant Sci. 2025 Jan 29;15:1480110. doi: 10.3389/fpls.2024.1480110 (PMC11813923; doi:10.3389/fpls.2024.1480110)
Supplement: Supplementary file 1 [file DataSheet1.docx]

Supplementary Figures

**Genotype by Environment Interactions and Phenotypic Traits Stability of the EUCLEG Faba Bean Collection**

**Dejan Sokolović^1^, Snežana Babić^1^, Mirjana Petrović^1*^, Ignacio Solís^2^, Mathias Cougnon^3^, Natalia Gutierrez^4^, Pertti Pärssinen^5^, Dirk Recheul^6^, Jasmina Radović^1^, Ana M. Torres^4^**

*** Correspondence:** Dejan Sokolović, dejan.sokolovic@ikbks.com


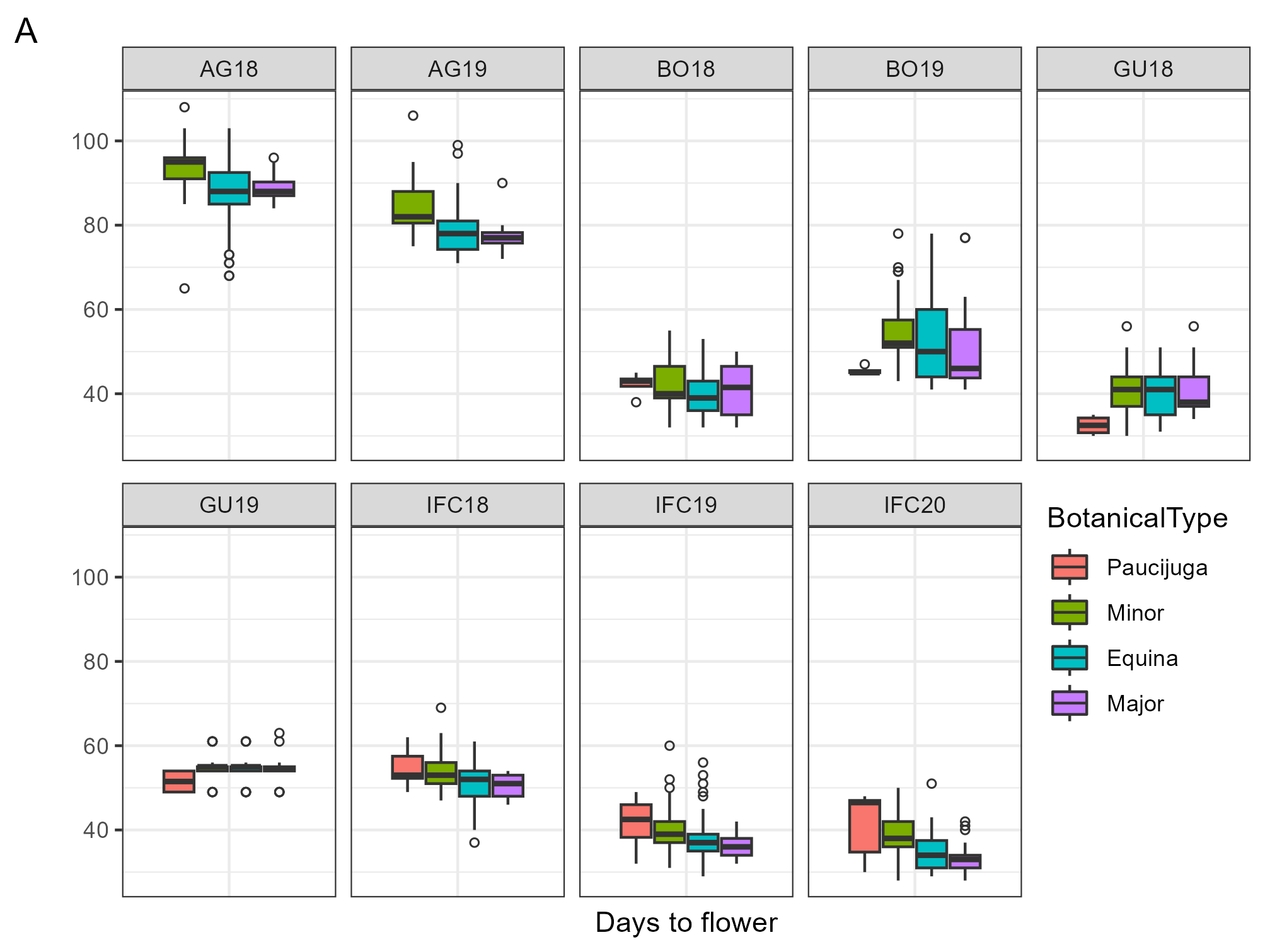


**Supplementary Figure S1a**: Boxplots of days to flower for nine environments, environmental acronyms begin with two or three letters identifying the trail location (AG- Agrovegetal Spain, IFC Institute for forage crops Kruševac Serbia, BO – Boreal Finland, and GU – Ghent Belgium) followed by the year) and botanical type (paucijuga, minor, equina and major).


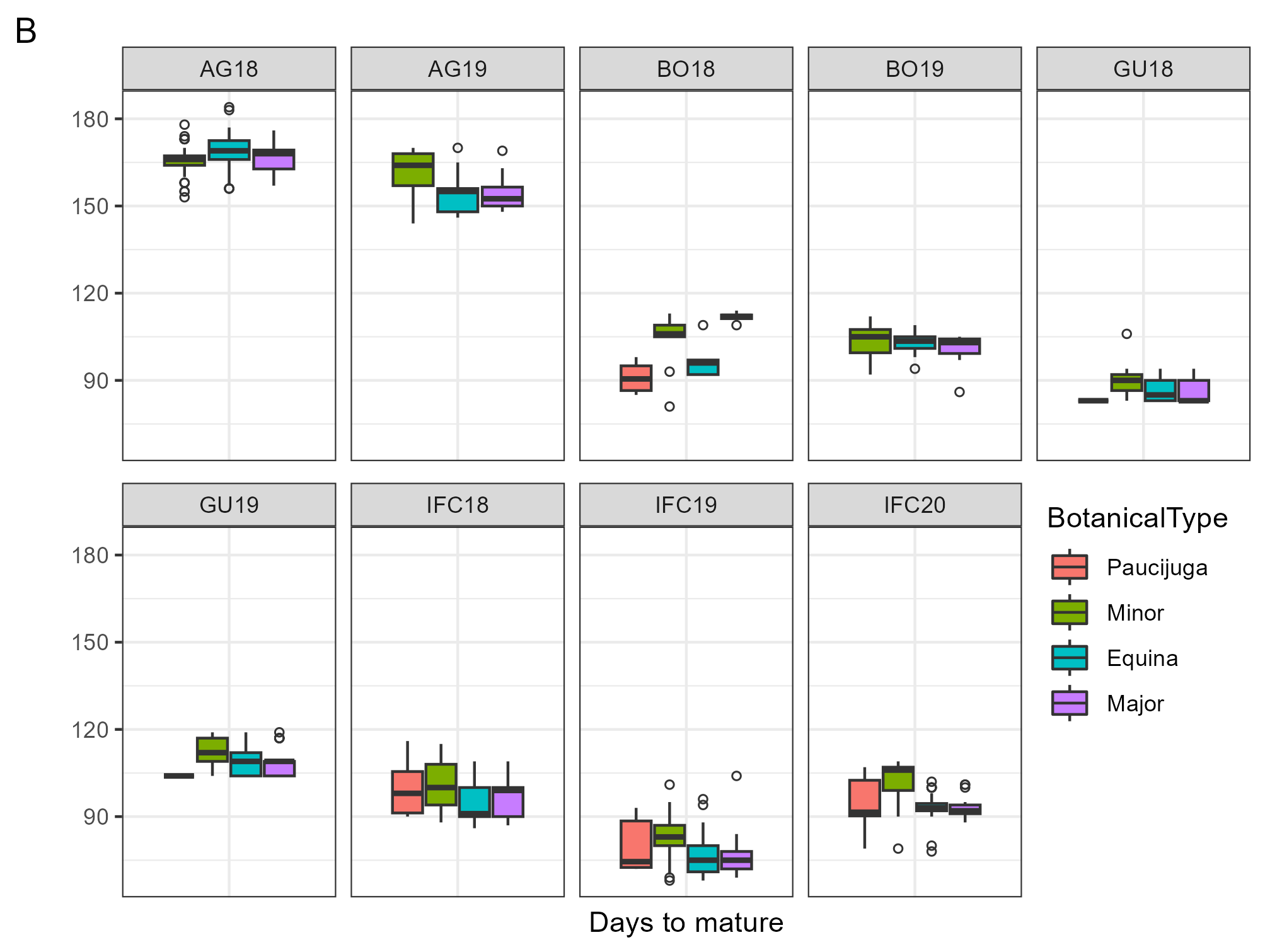


**Supplementary Figure S1b**: Boxplots of days to mature for nine environments, environmental acronyms begin with two or three letters identifying the trail location (AG- Agrovegetal Spain, IFC Institute for forage crops Kruševac Serbia, BO – Boreal Finland, and GU – Ghent Belgium) followed by the year) and botanical type (paucijuga, minor, equina and major).


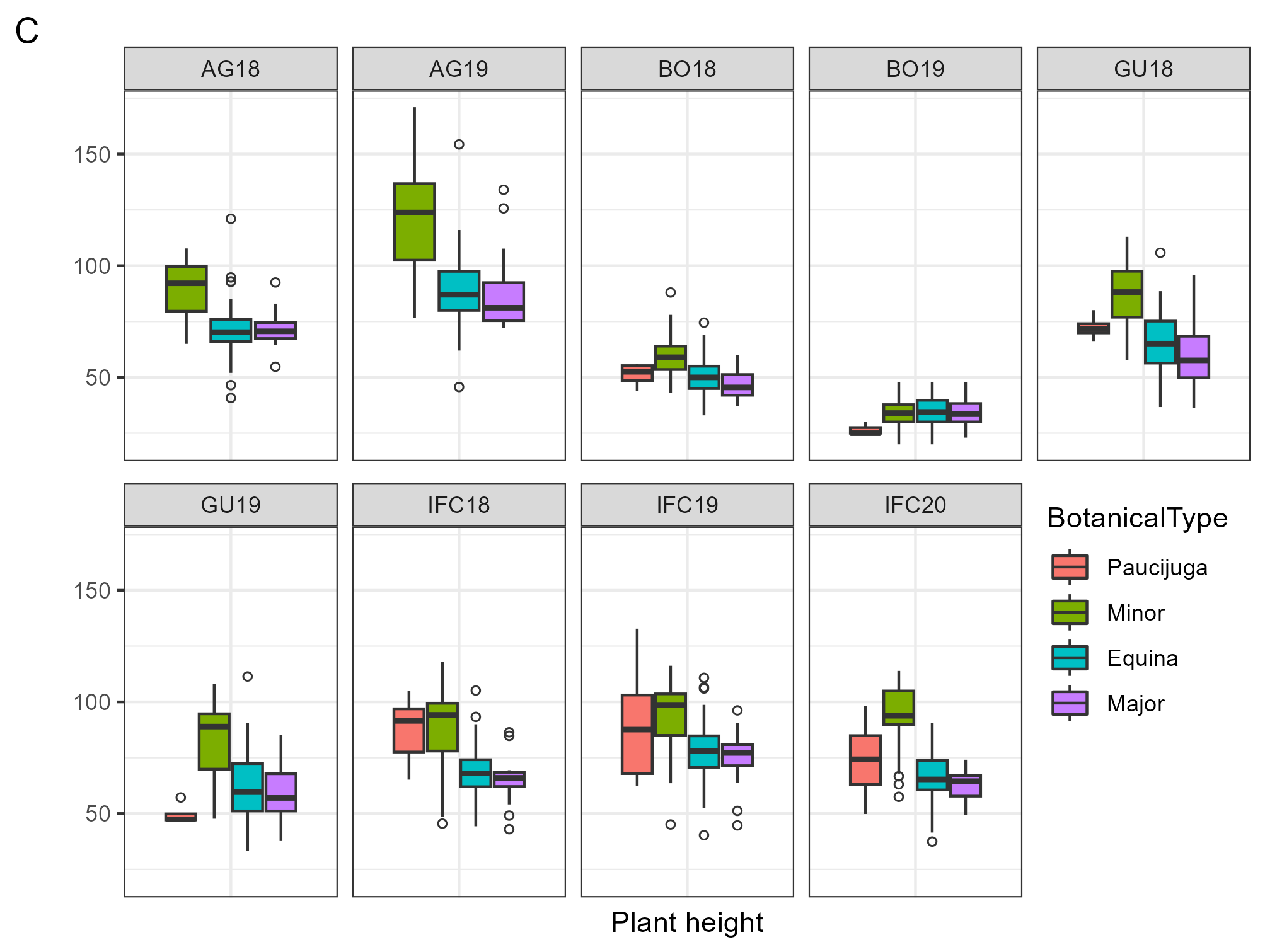


**Supplementary Figure S1c**: Boxplots of plant height for nine environments, environmental acronyms begin with two or three letters identifying the trail location (AG- Agrovegetal Spain, IFC Institute for forage crops Kruševac Serbia, BO – Boreal Finland, and GU – Ghent Belgium) followed by the year) and botanical type (paucijuga, minor, equina and major).


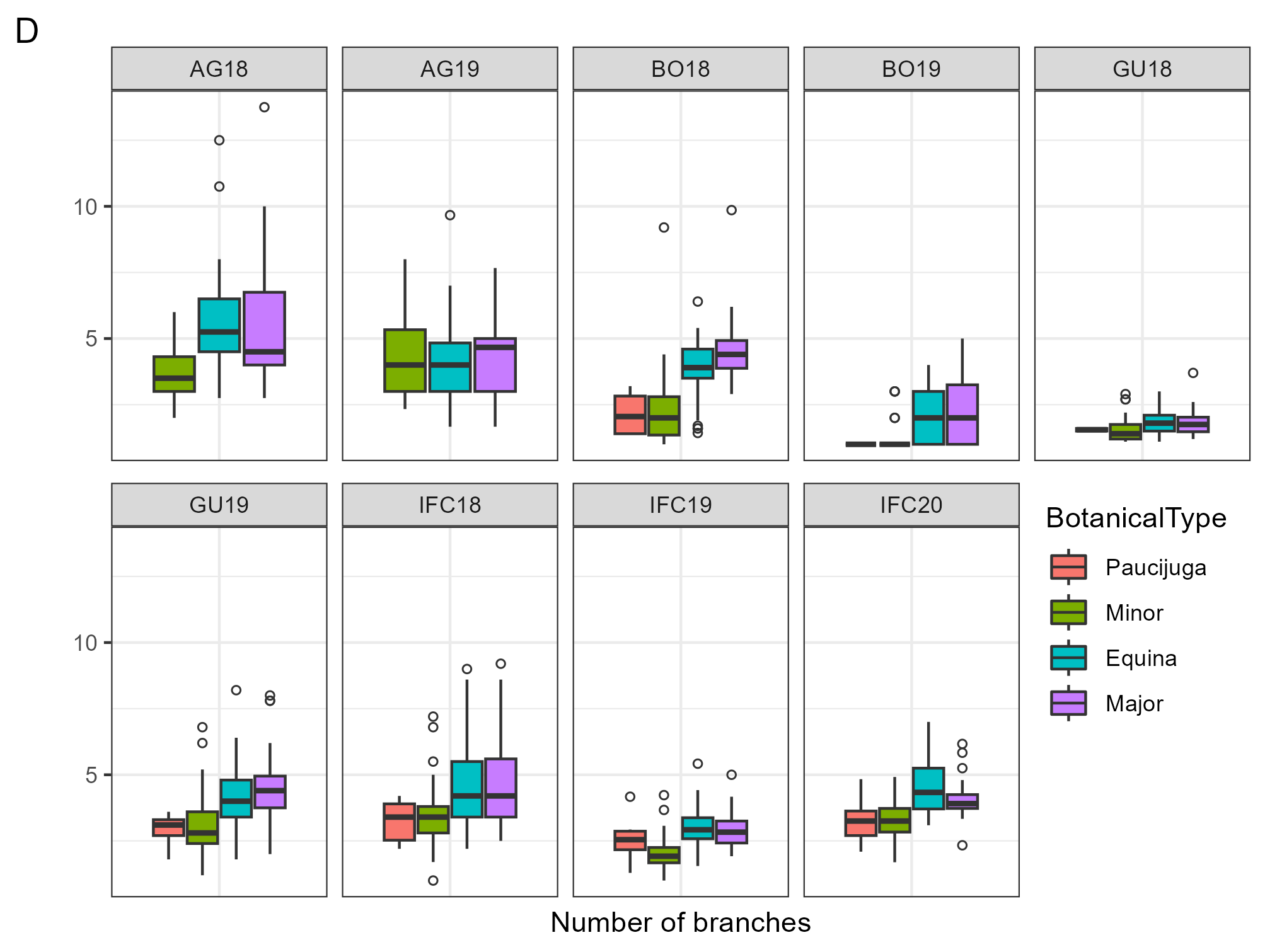


**Supplementary Figure S1d**: Boxplots of number of branches for nine environments, environmental acronyms begin with two or three letters identifying the trail location (AG- Agrovegetal Spain, IFC Institute for forage crops Kruševac Serbia, BO – Boreal Finland, and GU – Ghent Belgium) followed by the year) and botanical type (paucijuga, minor, equina and major).


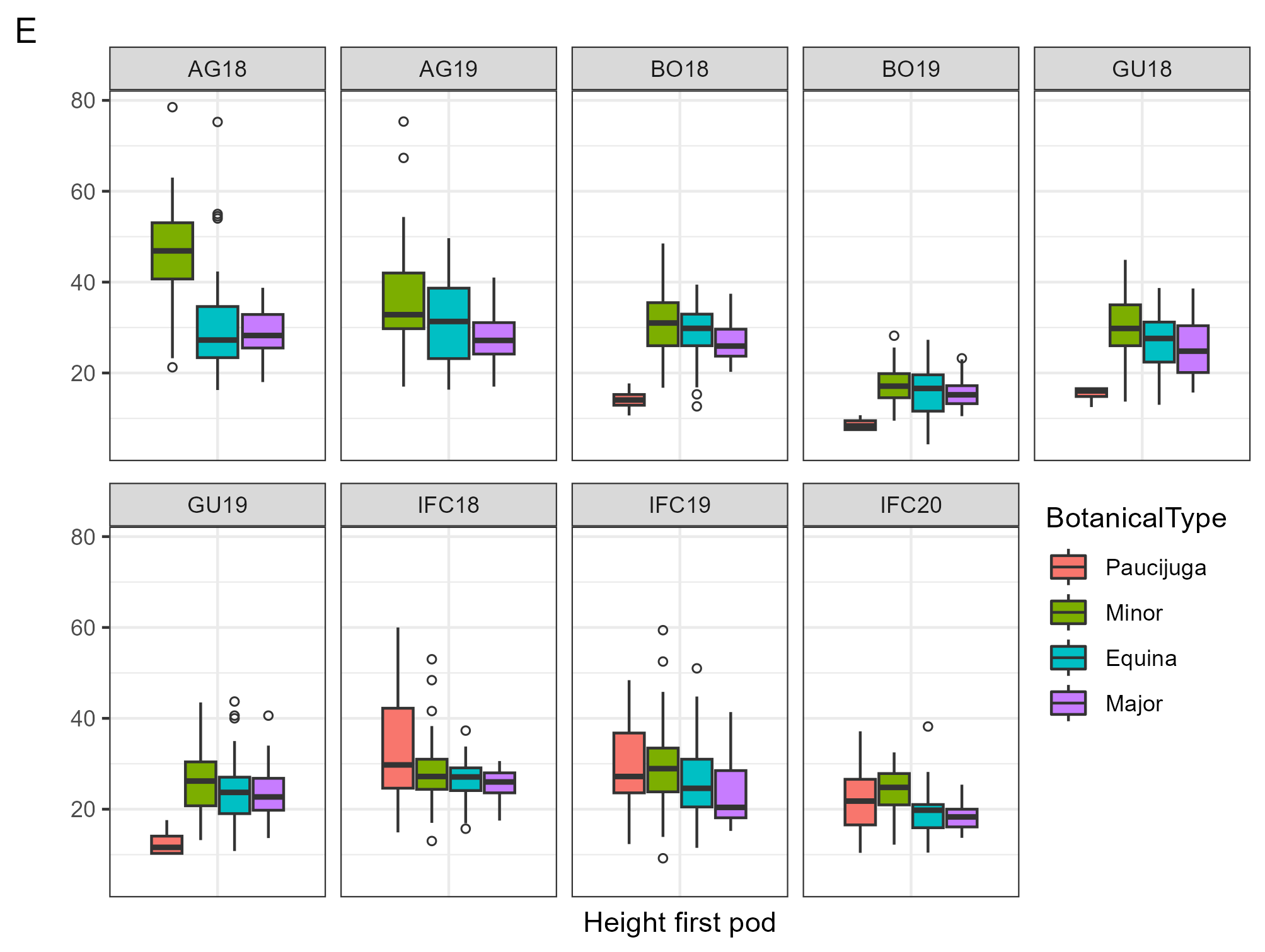


**Supplementary Figure S1e**: Boxplots of height of first pod for nine environments, environmental acronyms begin with two or three letters identifying the trail location (AG- Agrovegetal Spain, IFC Institute for forage crops Kruševac Serbia, BO – Boreal Finland, and GU – Ghent Belgium) followed by the year) and botanical type (paucijuga, minor, equina and major).


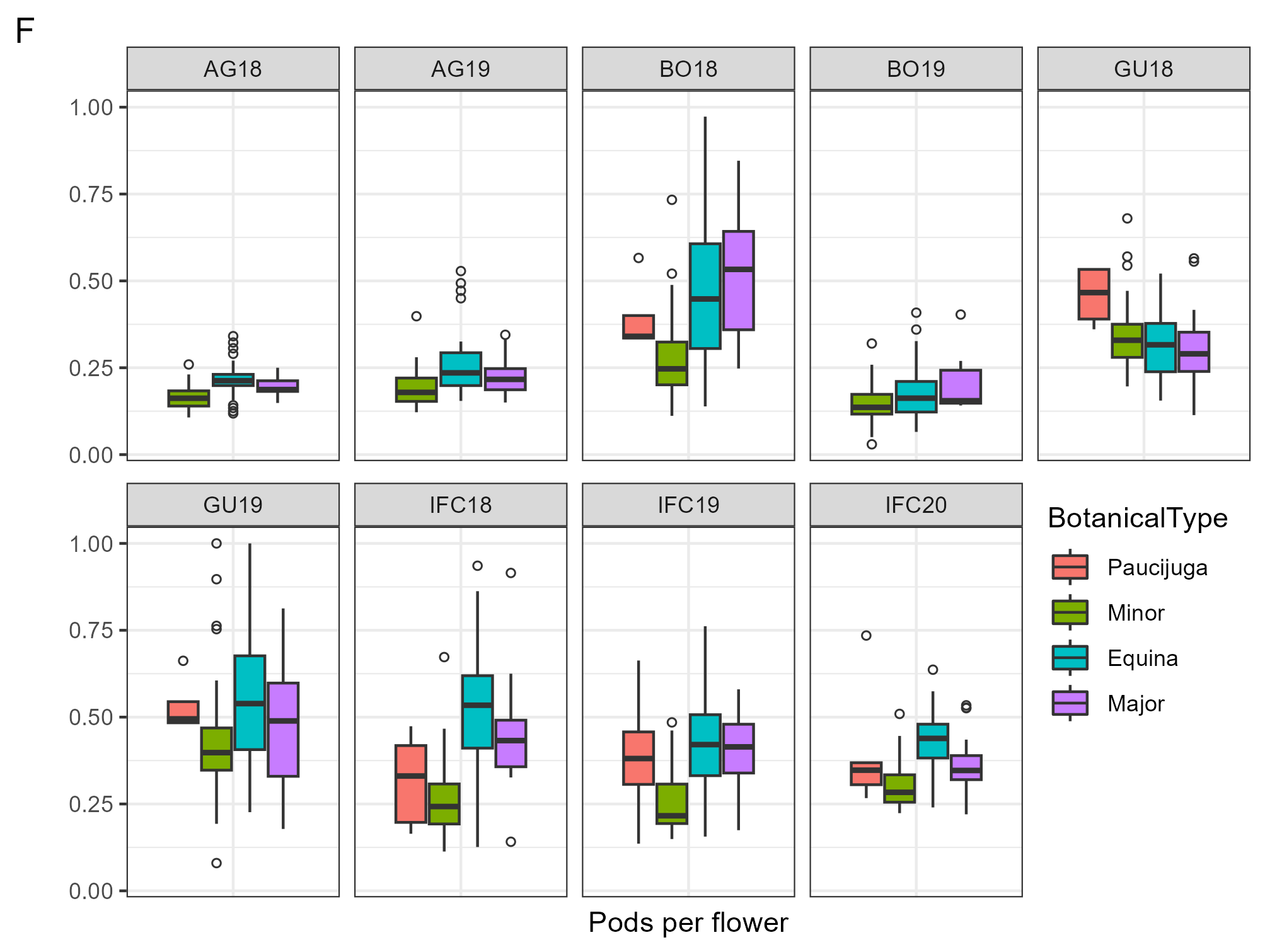


**Supplementary Figure S1f**: Boxplots of pods per flower for nine environments, environmental acronyms begin with two or three letters identifying the trail location (AG- Agrovegetal Spain, IFC Institute for forage crops Kruševac Serbia, BO – Boreal Finland, and GU – Ghent Belgium) followed by the year) and botanical type (paucijuga, minor, equina and major).


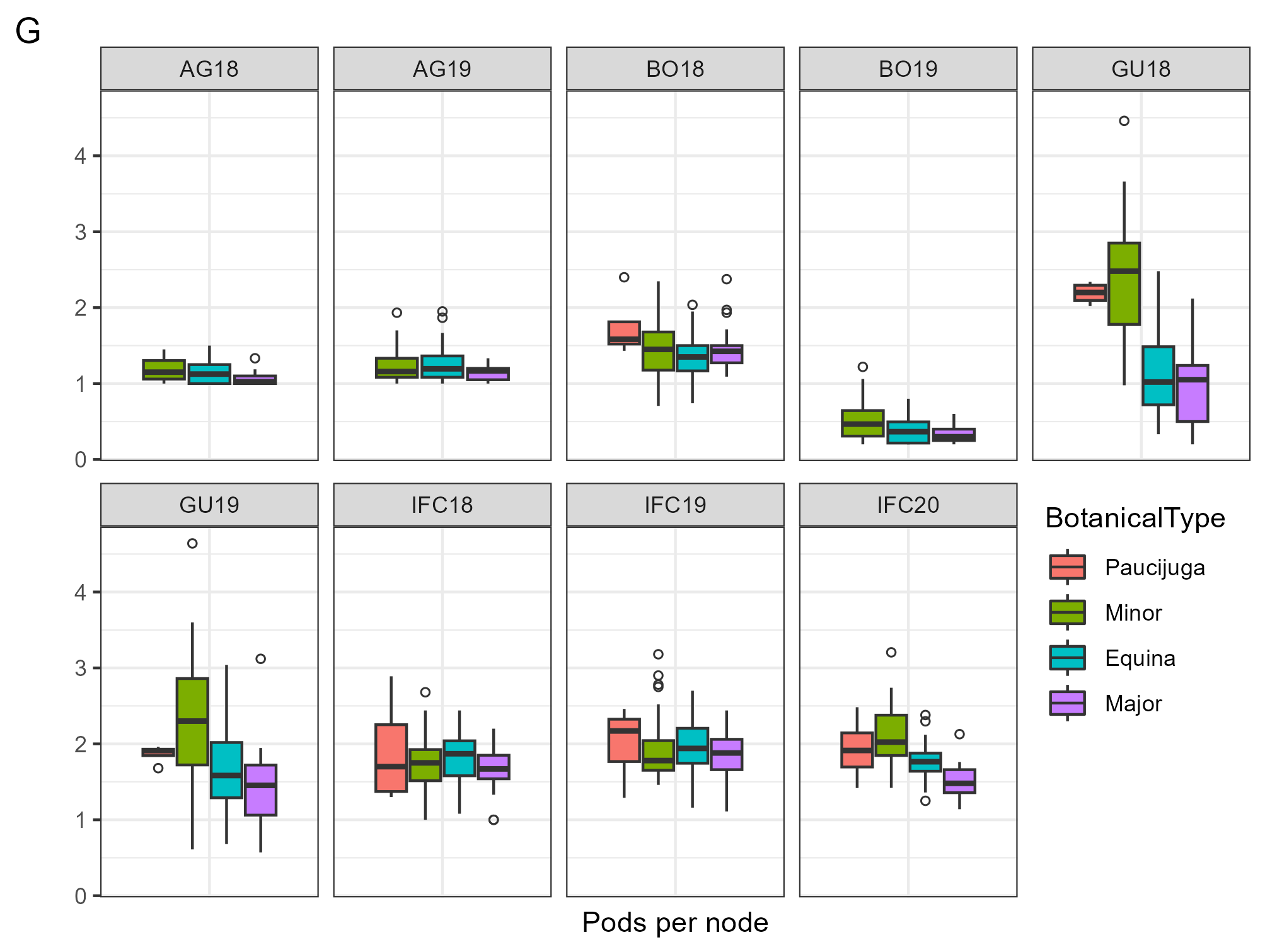


**Supplementary Figure S1g**: Boxplots of pods per node for nine environments, environmental acronyms begin with two or three letters identifying the trail location (AG- Agrovegetal Spain, IFC Institute for forage crops Kruševac Serbia, BO – Boreal Finland, and GU – Ghent Belgium) followed by the year) and botanical type (paucijuga, minor, equina and major).


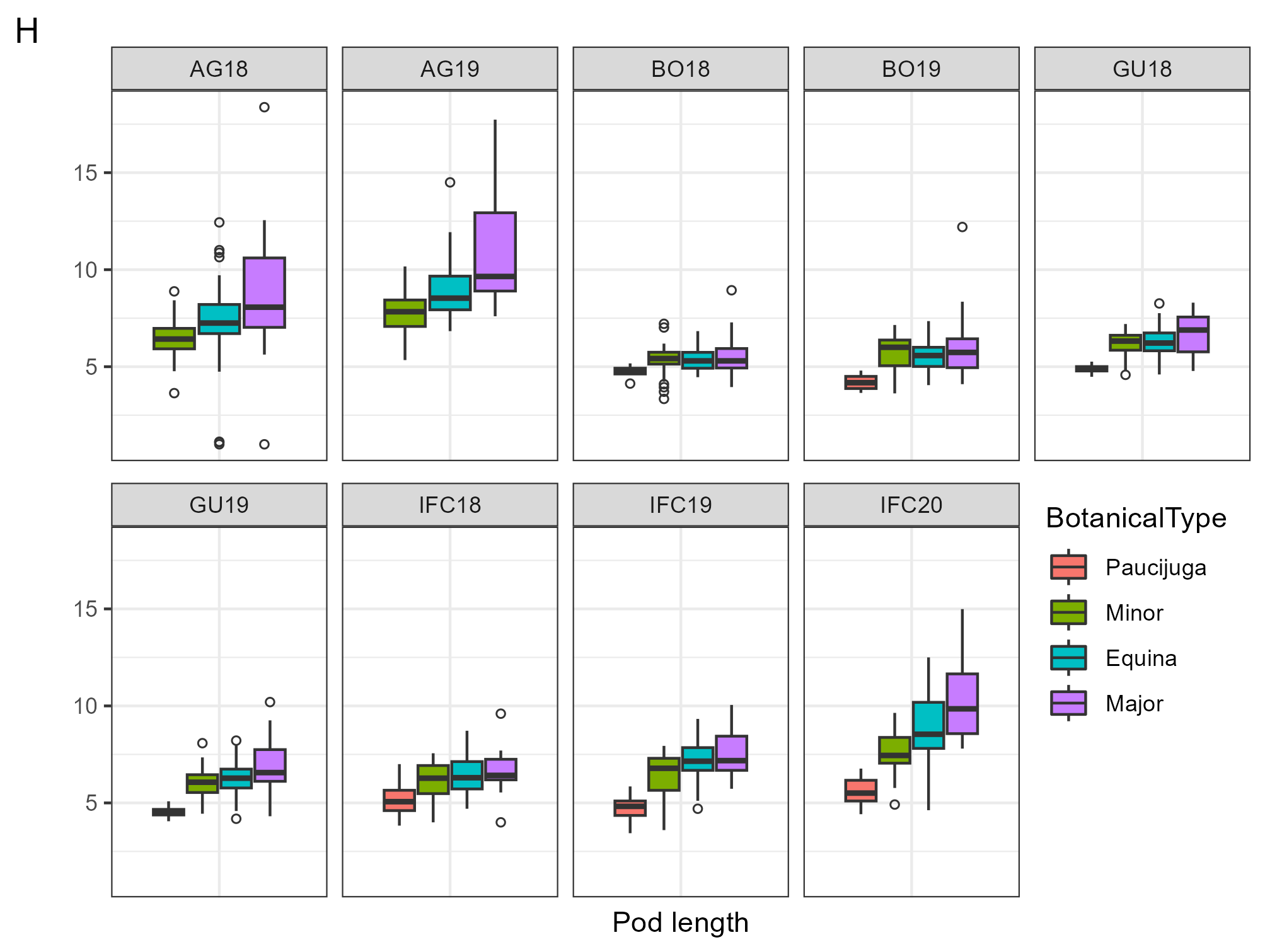


**Supplementary Figure S1h**: Boxplots of pod lenght for nine environments, environmental acronyms begin with two or three letters identifying the trail location (AG- Agrovegetal Spain, IFC Institute for forage crops Kruševac Serbia, BO – Boreal Finland, and GU – Ghent Belgium) followed by the year) and botanical type (paucijuga, minor, equina and major).


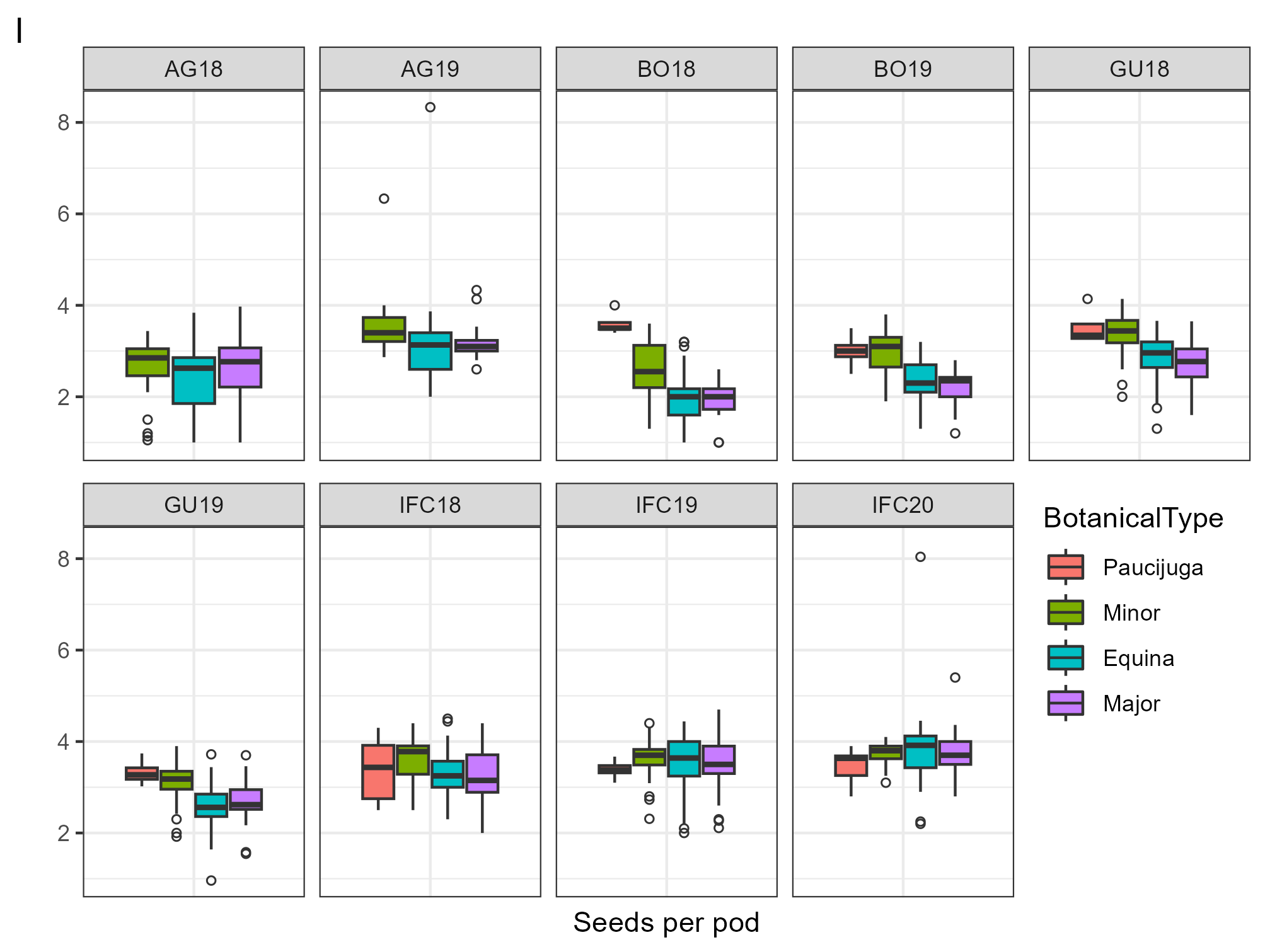


**Supplementary Figure S1i**: Boxplots of seeds per pod for nine environments, environmental acronyms begin with two or three letters identifying the trail location (AG- Agrovegetal Spain, IFC Institute for forage crops Kruševac Serbia, BO – Boreal Finland, and GU – Ghent Belgium) followed by the year) and botanical type (paucijuga, minor, equina and major).


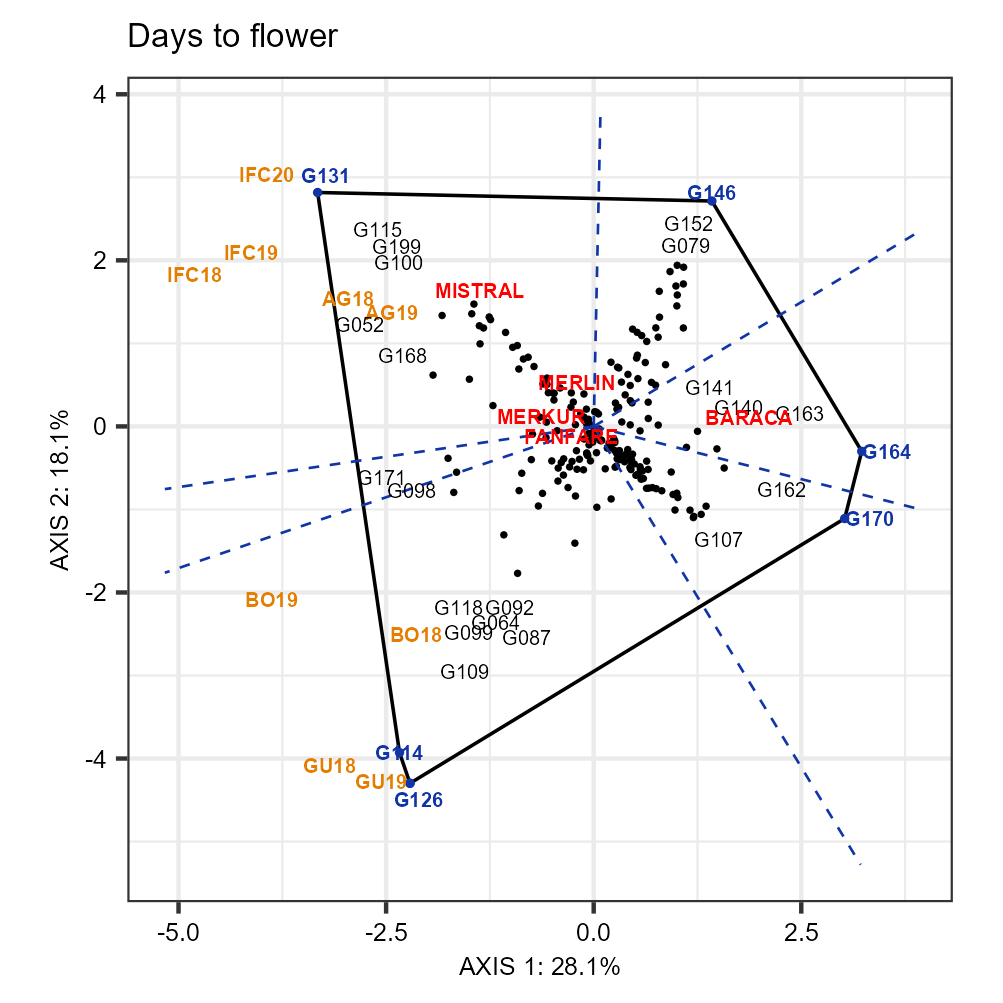


**Supplementary Figure S2A**: "Which-won-where" pattern of GGE biplot polygon view displaying the G + GE effect of 220 *Faba bean* genotypes in 9 environments, environmental acronyms begin with two or three letters identifying the trail location (AG- Agrovegetal Spain, IFC Institute for forage crops Kruševac Serbia, BO – Boreal Finland, and GU –Ghent Belgium) followed by the year) in 4 locations for days to flower. The biplots were based on centering = 0, SVP = 2, and scaling = 0.


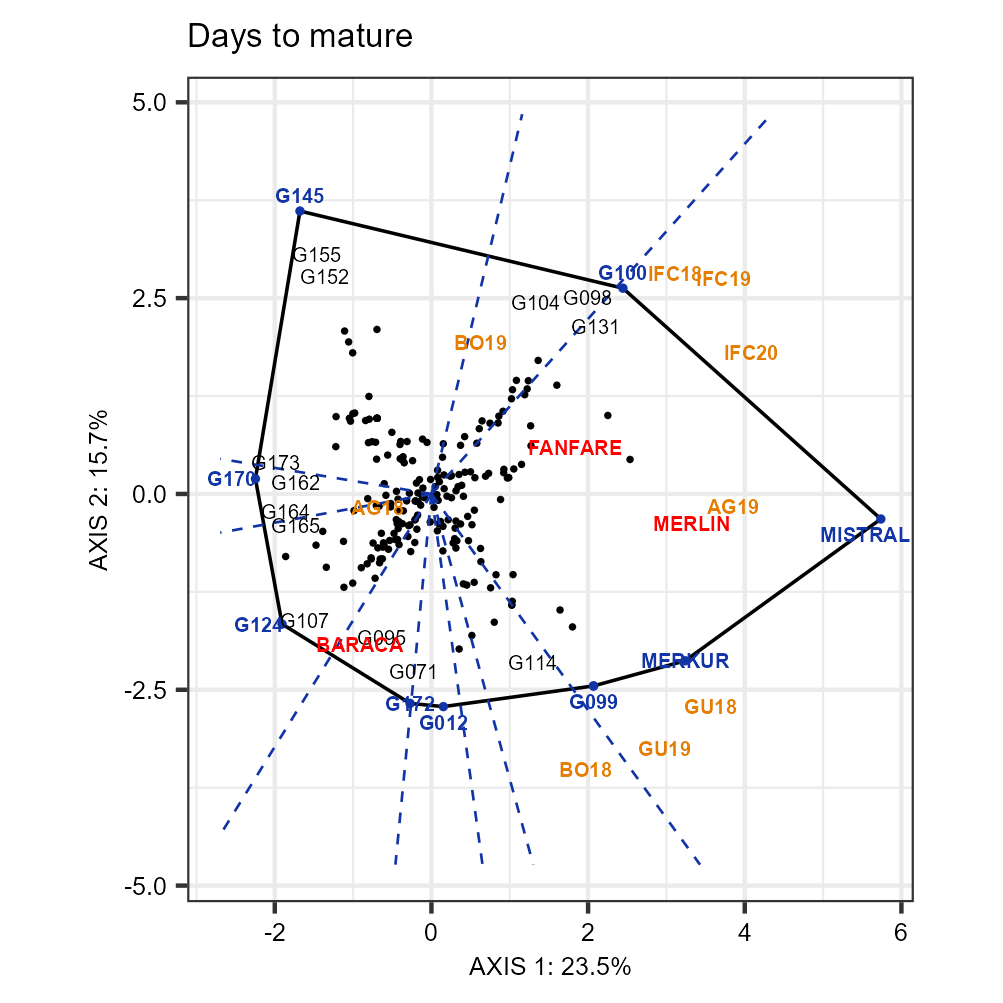


**Supplementary Figure S2B**: "Which-won-where" pattern of GGE biplot polygon view displaying the G + GE effect of 220 *Faba bean* genotypes in 9 environments, environmental acronyms begin with two or three letters identifying the trail location (AG- Agrovegetal Spain, IFC Institute for forage crops Kruševac Serbia, BO – Boreal Finland, and GU –Ghent Belgium) followed by the year) in 4 locations for days to mature. The biplots were based on centering = 0, SVP = 2, and scaling = 0.


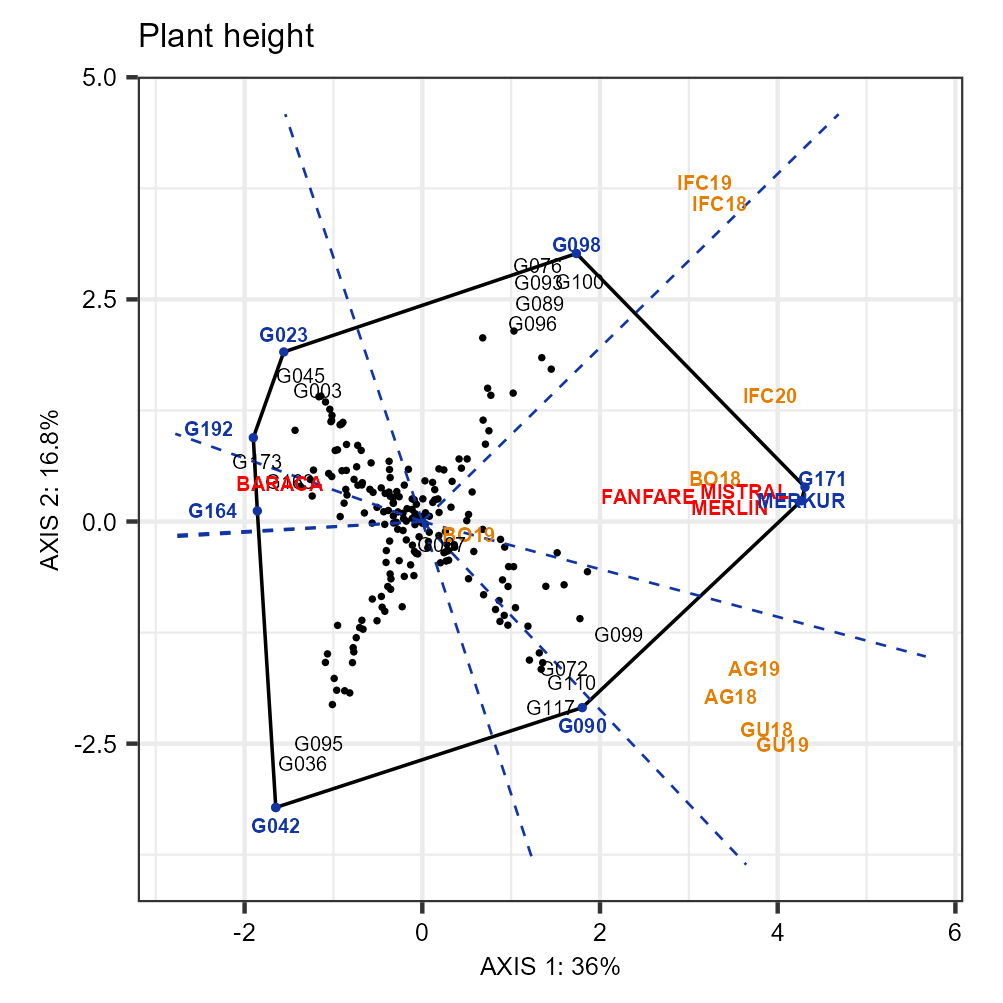


**Supplementary Figure S2C**: "Which-won-where" pattern of GGE biplot polygon view displaying the G + GE effect of 220 *Faba bean* genotypes in 9 environments, environmental acronyms begin with two or three letters identifying the trail location (AG- Agrovegetal Spain, IFC Institute for forage crops Kruševac Serbia, BO – Boreal Finland, and GU –Ghent Belgium) followed by the year) in 4 locations for plant height. The biplots were based on centering = 0, SVP = 2, and scaling = 0.


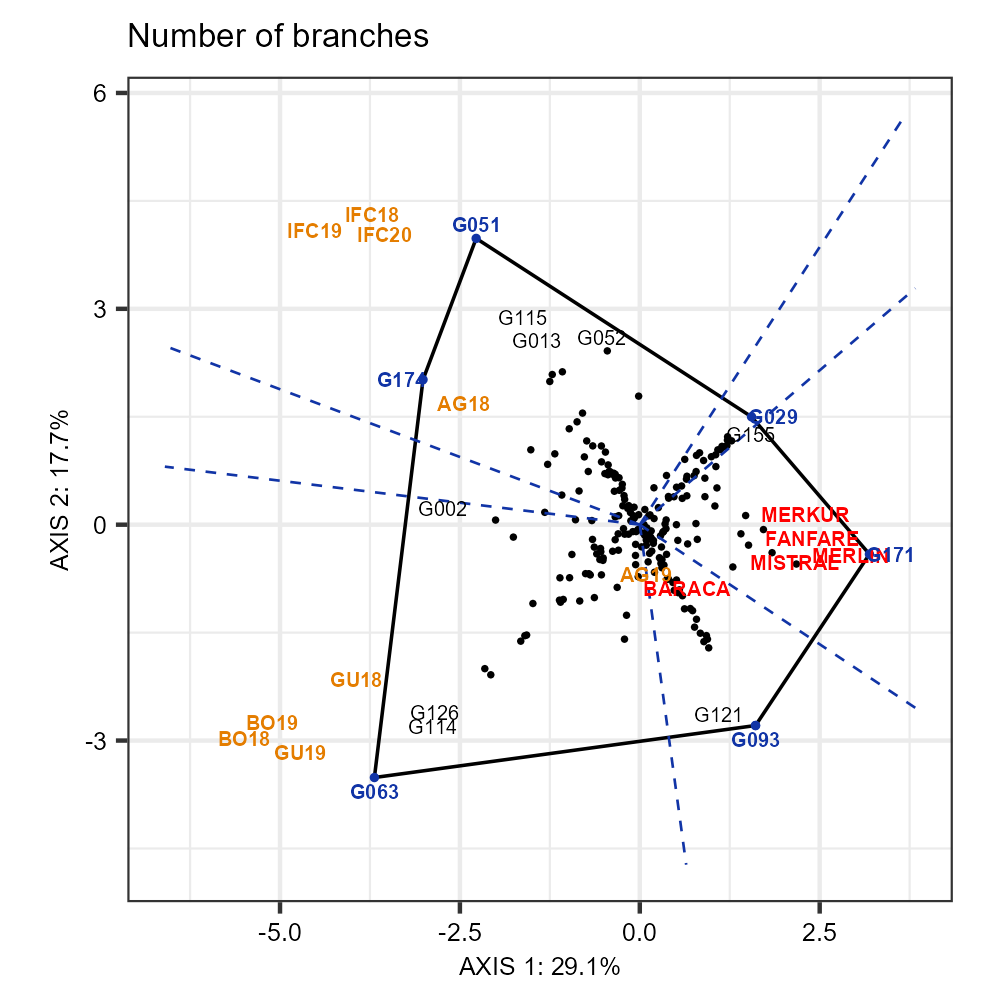


**Supplementary Figure S2D**: "Which-won-where" pattern of GGE biplot polygon view displaying the G + GE effect of 220 *Faba bean* genotypes in 9 environments, environmental acronyms begin with two or three letters identifying the trail location (AG- Agrovegetal Spain, IFC Institute for forage crops Kruševac Serbia, BO – Boreal Finland, and GU –Ghent Belgium) followed by the year) in 4 locations for number of branches. The biplots were based on centering = 0, SVP = 2, and scaling = 0.


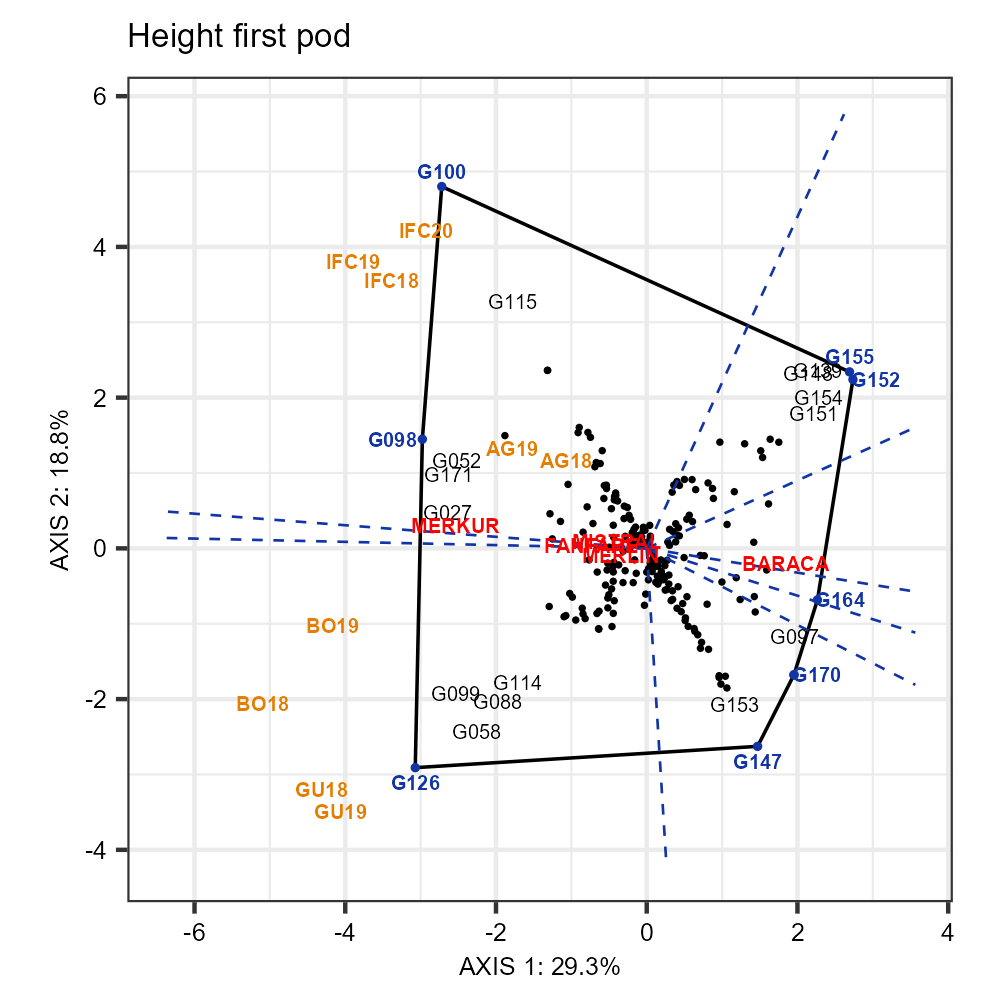


**Supplementary Figure S2E**: "Which-won-where" pattern of GGE biplot polygon view displaying the G + GE effect of 220 *Faba bean* genotypes in 9 environments, environmental acronyms begin with two or three letters identifying the trail location (AG- Agrovegetal Spain, IFC Institute for forage crops Kruševac Serbia, BO – Boreal Finland, and GU –Ghent Belgium) followed by the year) in 4 locations for height first pod. The biplots were based on centering = 0, SVP = 2, and scaling = 0.


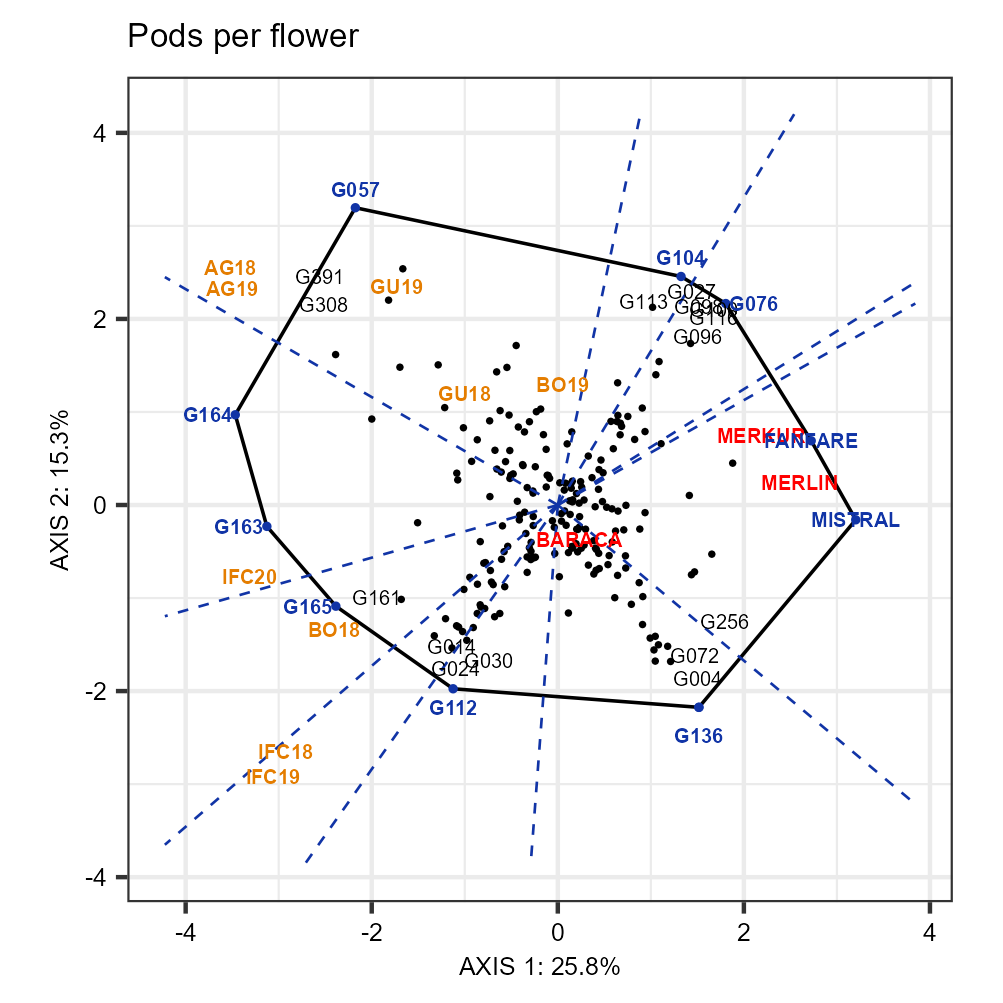


**Supplementary Figure S2F**: "Which-won-where" pattern of GGE biplot polygon view displaying the G + GE effect of 220 *Faba bean* genotypes in 9 environments, environmental acronyms begin with two or three letters identifying the trail location (AG- Agrovegetal Spain, IFC Institute for forage crops Kruševac Serbia, BO – Boreal Finland, and GU –Ghent Belgium) followed by the year) in 4 locations for pods per flower. The biplots were based on centering = 0, SVP = 2, and scaling = 0.


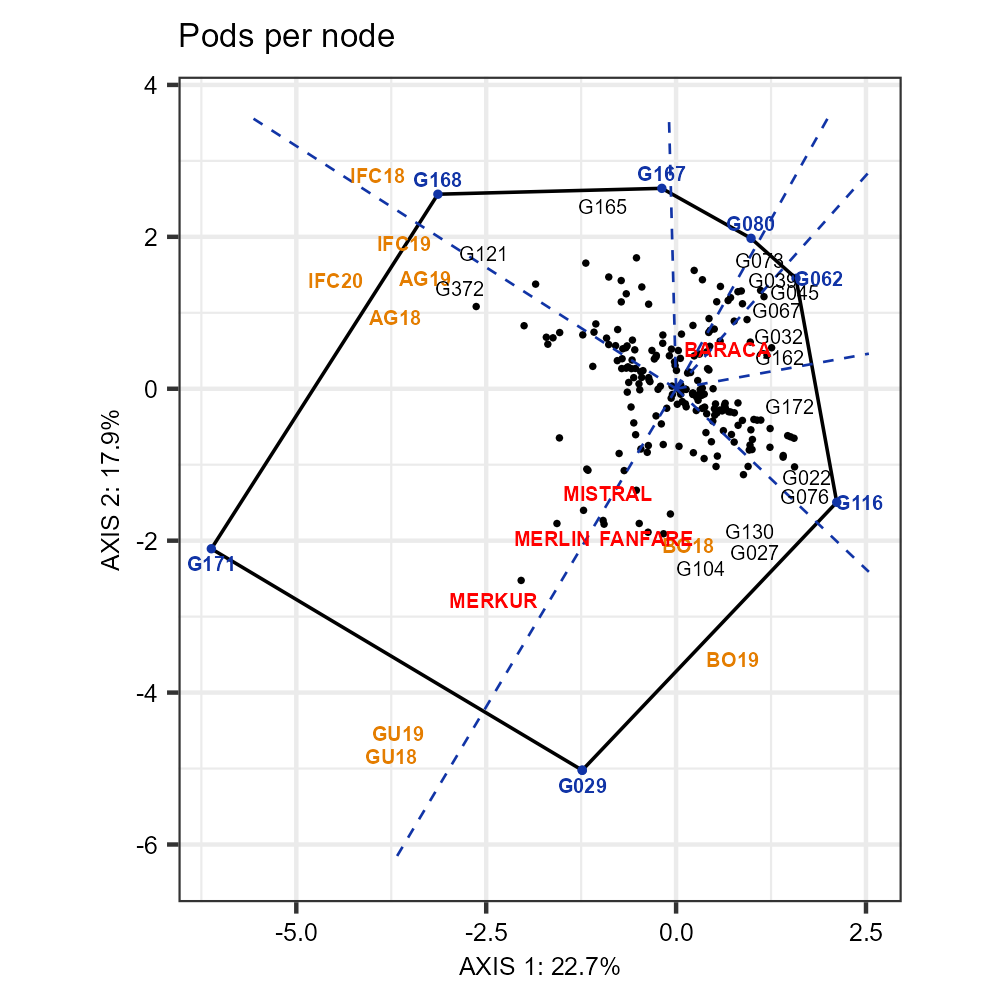


**Supplementary Figure S2 G**: "Which-won-where" pattern of GGE biplot polygon view displaying the G + GE effect of 220 *Faba bean* genotypes in 9 environments, environmental acronyms begin with two or three letters identifying the trail location (AG- Agrovegetal Spain, IFC Institute for forage crops Kruševac Serbia, BO – Boreal Finland, and GU –Ghent Belgium) followed by the year) in 4 locations for pods per node. The biplots were based on centering = 0, SVP = 2, and scaling = 0.


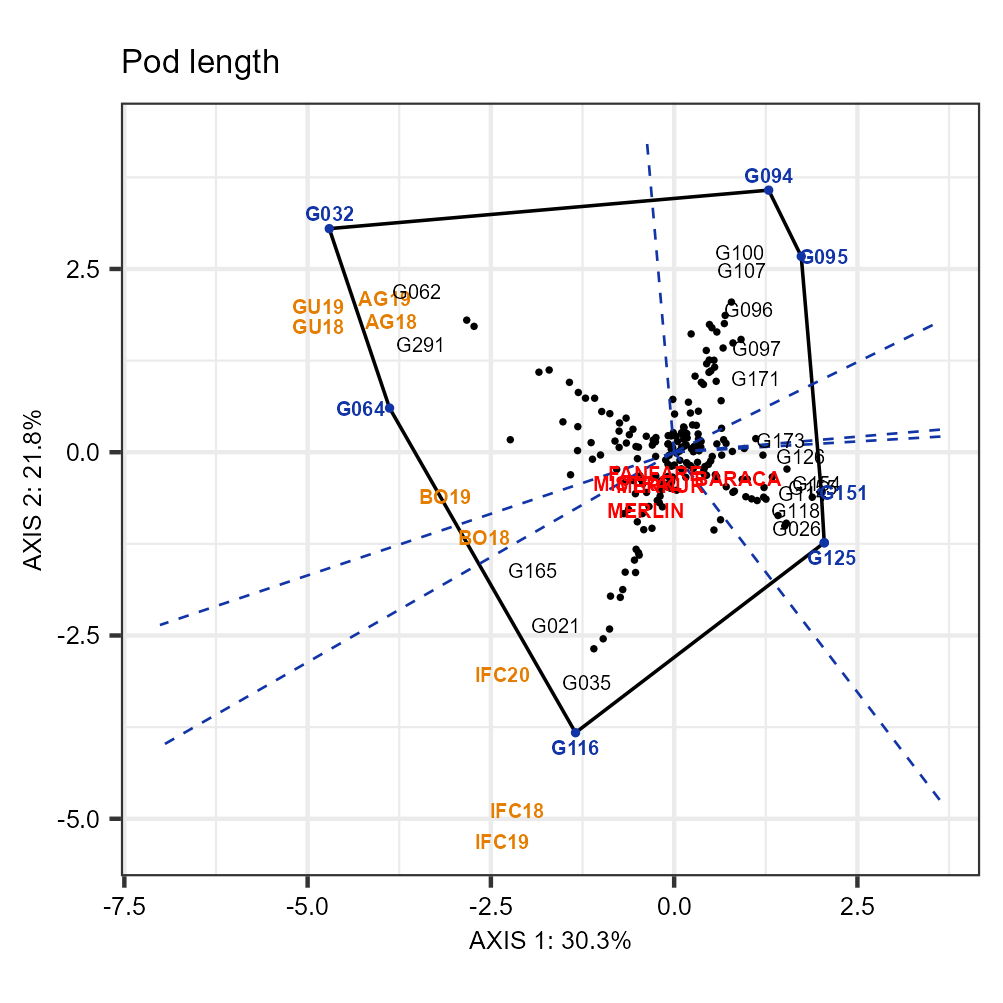


**Supplementary Figure S2H**: "Which-won-where" pattern of GGE biplot polygon view displaying the G + GE effect of 220 *Faba bean* genotypes in 9 environments, environmental acronyms begin with two or three letters identifying the trail location (AG- Agrovegetal Spain, IFC Institute for forage crops Kruševac Serbia, BO – Boreal Finland, and GU –Ghent Belgium) followed by the year) in 4 locations for pod length. The biplots were based on centering = 0, SVP = 2, and scaling = 0.


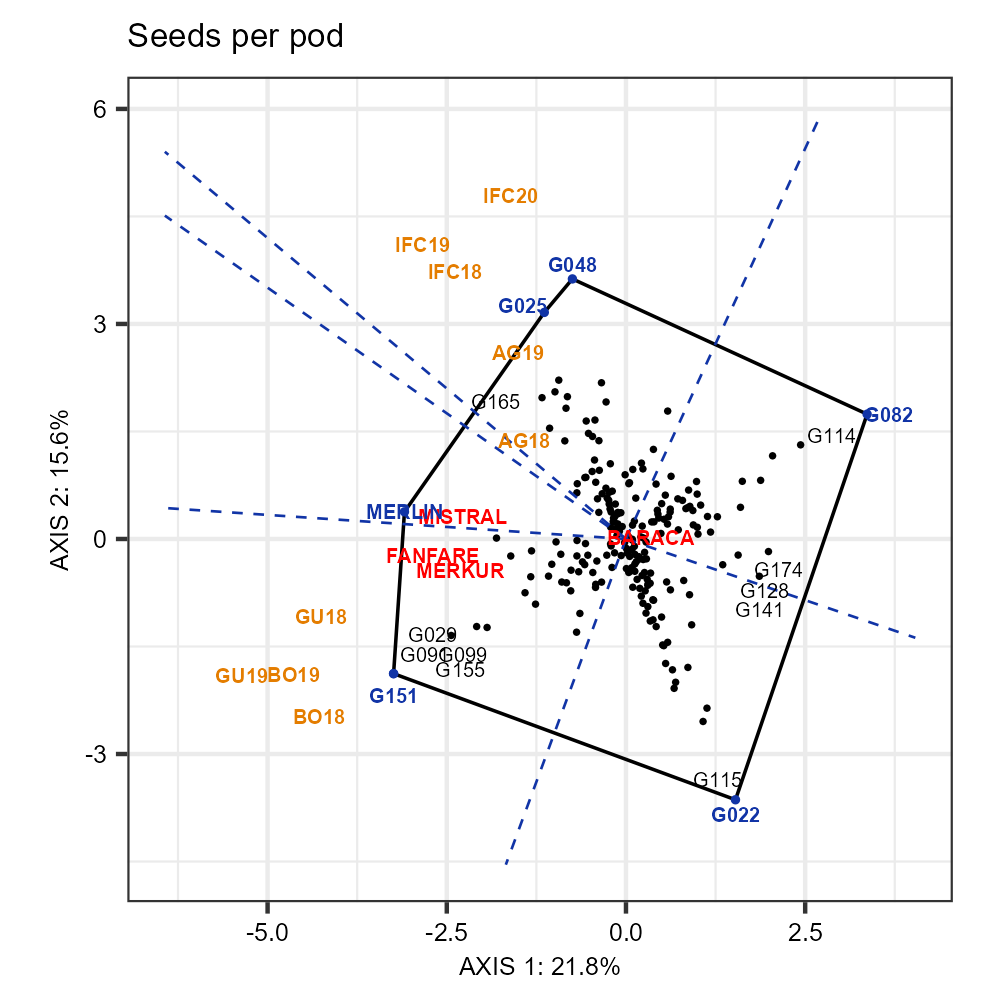


**Supplementary Figure S2I**: "Which-won-where" pattern of GGE biplot polygon view displaying the G + GE effect of 220 *Faba bean* genotypes in 9 environments, environmental acronyms begin with two or three letters identifying the trail location (AG- Agrovegetal Spain, IFC Institute for forage crops Kruševac Serbia, BO – Boreal Finland, and GU –Ghent Belgium) followed by the year) in 4 locations for seds per pod. The biplots were based on centering = 0, SVP = 2, and scaling = 0.
